# Recent Advances in In Vivo Somatic Cell Gene Modification in Newborn Pups

**DOI:** 10.3390/ijms242015301

**Published:** 2023-10-18

**Authors:** Shingo Nakamura, Kazunori Morohoshi, Emi Inada, Yoko Sato, Satoshi Watanabe, Issei Saitoh, Masahiro Sato

**Affiliations:** 1 Division of Biomedical Engineering, National Defense Medical College Research Institute, Tokorozawa 359-8513, Japan; kmorohoshi@ndmc.ac.jp; 2 Department of Pediatric Dentistry, Graduate School of Medical and Dental Sciences, Kagoshima University, Kagoshima 890-8544, Japan; inada@dent.kagoshima-u.ac.jp; 3 Graduate School of Public Health, Shizuoka Graduate University of Public Health, Aoi-ku, Shizuoka 420-0881, Japan; sato.yoko.shiz@gmail.com; 4 Institute of Livestock and Grassland Science, NARO, Tsukuba 305-0901, Japan; kettle@affrc.go.jp; 5 Department of Pediatric Dentistry, Asahi University School of Dentistry, Mizuho 501-0296, Japan; isaitoh@dent.asahi-u.ac.jp; 6 Department of Genome Medicine, National Center for Child Health and Development, Setagaya-ku, Tokyo 157-8535, Japan; sato-masa@ncchd.go.jp

**Keywords:** CRISPR/Cas9, genetically modified animal, genome editing, in vivo genome engineering, newborn, somatic mutation

## Abstract

Germline manipulation at the zygote stage using the CRISPR/Cas9 system has been extensively employed for creating genetically modified animals and maintaining established lines. However, this approach requires a long and laborious task. Recently, many researchers have attempted to overcome these limitations by generating somatic mutations in the adult stage through tail vein injection or local administration of CRISPR reagents, as a new strategy called “in vivo somatic cell genome editing”. This approach does not require manipulation of early embryos or strain maintenance, and it can test the results of genome editing in a short period. The newborn is an ideal stage to perform in vivo somatic cell genome editing because it is immune-privileged, easily accessible, and only a small amount of CRISPR reagents is required to achieve somatic cell genome editing throughout the entire body, owing to its small size. In this review, we summarize in vivo genome engineering strategies that have been successfully demonstrated in newborns. We also report successful in vivo genome editing through the neonatal introduction of genome editing reagents into various sites in newborns (as exemplified by intravenous injection via the facial vein), which will be helpful for creating models for genetic diseases or treating many genetic diseases.

## 1. Introduction

Genetic manipulation in vivo using viral or non-viral vectors has been recognized as a useful and powerful tool for elucidating the role of a gene of interest (GOI), creating genetically modified animals, and rescuing genetic defects caused by mutations in genes that are important for individual life.

Germline manipulation is based on gene delivery to early embryos, such as fertilized eggs (zygotes), through pronuclear microinjection of nucleic acids, electroporation (EP) in the presence of nucleic acids, or transduction in the presence of viral vectors [1,2]. In most cases, these are performed using zygotes in vitro, which have been isolated from the ampulla region of an oviduct in a pregnant female. To allow these manipulated embryos to develop further, egg transfer (ET) to the recipient female reproductive tract (in a pseudopregnant state), such as the oviduct or uterus, is a prerequisite. However, the preparation of the recipient female is laborious, and ET itself requires special skills. To avoid such ex vivo handling of zygotes, novel techniques, such as Genome Editing via Oviductal Nucleic Acids Delivery (GONAD) or improved GONAD (*i*-GONAD) [3,4], which enable in situ genome editing of zygotes without the need to isolate zygotes, have been developed. These do not require the ex vivo handling of embryos and are performed solely in vivo (within the oviductal lumen of a pregnant female). Breath-controlled micropipette-based instillation of genome editing components into the oviductal lumen of a pregnant female on day 0 or 1 of pregnancy (day 0 of pregnancy is defined as the day when the vaginal plug is recognized in the morning) takes place under a dissecting microscope, followed by EP towards the entire oviduct. Notably, in this case, it does not require any recipient females, which are usually needed for the ET of ex vivo manipulated embryos. In summary, the creation of knockout (KO) or transgenic (Tg) animals is still recognized as a standard approach for studying the function of a GOI in vivo. However, systematic functional screening of candidate genes and maintenance of established lines are time-consuming and costly. Moreover, various compensatory mechanisms often make it difficult to interpret the results.

In contrast to the germline manipulation, a new strategy called “in vivo somatic cell genome editing” [5] or “somatic-Tg mice” [6] is emerging as an alternative to the abovementioned germline manipulation, which generates somatic gene modification in the fetal stage through in utero injection of nucleic acids, or in the adult stage through tail vein injection or local administration of nucleic acids. This approach does not require manipulation of early embryos or strain maintenance, and it can test the results of genome editing in a short period. However, in the case of in utero genetic manipulation, surgery on a pregnant female is always required, which often causes fetal death after surgery and requires special skills to introduce nucleic acids to the target site of a fetus under a dissecting microscope [7]. For in vivo gene manipulation in the adult stage, a tail-vein-based gene introduction approach requires a large amount of nucleic acids to achieve somatic cell genome editing throughout the entire body. Furthermore, it often causes immunological rejection of certain types of nucleic acids (as exemplified by viral vectors) after repeated administration.

In this context, the newborn is an ideal stage to perform in vivo somatic cell genome modification (including genome editing and transgenesis), since it is immune-privileged, easily accessible, and only a small amount of genome editing reagents is required to achieve somatic cell genome engineering throughout the body, owing to its small size. This review provides a comprehensive summary of in vivo genome engineering strategies that are applicable to newborn animals. Notably, genome editing techniques are already widely used in research on adult animals, but there are still only a few cases of gene editing of targeted newborns. We report successful in vivo genome editing outcomes achieved through the introduction of genome editing reagents at various sites in newborns. This approach will aid in developing genetic disease models and treating many genetic diseases.

## 2. Concept for In Vivo Organ/Tissue Genome Editing

Genome editing, as exemplified by the CRISPR/CRISPR-associated protein 9 (Cas9) (CRISPR/Cas9) system, has been well established as a useful genome engineering tool that enables researchers to examine gene function, create animal models of human diseases, modulate immunological systems and carcinogenesis, suppress viral infections, and improve genetically transformed organisms [8].

The CRISPR/Cas9 system employs a bacterial-derived Cas9 endonuclease and short guide RNA (gRNA), which is complementary to the target site in the genome [9]. When these two components are introduced into a cell, they form a complex called ribonucleoprotein (RNP), which binds to a specific chromosomal locus. After RNP binding, double-stranded (ds) DNA breaks (DSBs) occur only 3–4 bp in front of the protospacer adjacent motif (PAM) [9]. The two edges generated after the DSB are repaired through the DNA repair machinery of the cell, namely, non-homologous end-joining (NHEJ), microhomology-mediated end-joining (MMEJ), and homology-directed repair (HDR) pathways. In the absence of a DNA donor, the two edges are rejoined through NHEJ, which often causes the alteration of nucleotides near the DSB site, leading to random insertions or deletions of nucleotides, called “indels”. In the case of MMEJ, it employs naturally occurring microhomology of 5–25 bp present on either side of the DSB to mediate end-joining [10]. The outcome of MMEJ is a reproducible deletion of intervening sequences while retaining one copy of the microhomology. For this reason, MMEJ is normally considered to be mutagenic, because of an overall loss of genetic information through precise deletion [11]. In the case of HDR, it requires a DNA donor (in which homologous regions exist on both sides), and the broken ends are repaired by homologous recombination using a DNA template. However, HDR’s efficiency is low, and it is only active in dividing cell types, limiting the range of proper HDR applications [9].

Notably, two classes of CRISPR/Cas9-mediated DNA base editors (BEs)—cytosine base editors (CBEs) and adenine base editors (ABEs)—have recently been developed to overcome these limitations [12,13,14]. BEs can directly install point mutations in cellular DNA without inducing DSBs. According to Kantor et al. [15], CBEs are targeted to a specific locus by a gRNA using Cas9 nickase (nCas9) or catalytically inactive “dead” Cas9 (dCas9) fused to a cytidine deaminase. CBEs can convert cytidine to uridine within a minute editing window near the PAM site. Uridine is converted to thymidine via base excision repair, creating a C-to-T or G-to-A change on the opposite DNA strand. ABEs have been engineered to convert adenosine to inosine, which is treated by the cell in a manner similar to guanosine, creating an A-to-G (or T-to-C) change. Adenine deaminases do not exist in nature but have been created by the directed evolution of *Escherichia coli* TadA, a tRNA adenine deaminase. Similar to the CBEs, the evolved TadA domain was fused to a Cas9 protein to create the ABE. Prime editors (PEs) are the latest additions to the CRISPR genome engineering toolkit. They use an engineered reverse transcriptase (RT) fused to Cas9 nickase and a prime editing guide RNA (pegRNA), which significantly differs from regular sgRNAs [16]. The pegRNA consists of two components: (a) a sequence complementary to the target sites that direct nCas9 to its target sequence, and (b) an additional sequence spelling the desired sequence changes [16]. The 5′ of the pegRNA binds to the primer binding site region on the DNA, resulting in the exposure of the non-complimentary strand. The unbound DNA of the PAM-containing strand is nicked by Cas9, creating an RT primer that is linked to nCas9. The nicked PAM strand is then extended by RT using the interior of the pegRNA as a template, consequently modifying the target region in a programmable manner [16]. BEs and PEs are effective tools for enabling precise nucleotide substitutions in a programmable manner, without requiring DSBs or a donor template. Notably, both BEs and PEs have remarkable potential as therapeutic tools for correcting disease-causing mutations in the human genome [15]. For example, Song et al. [17] demonstrated the successful delivery of RNA-encoded ABEs into the livers of tyrosinemia type 1 model mice, thereby correcting splice-variant mutations and rescuing the phenotype.

Genome editing of germline cells (i.e., primordial germ cells, gamete progenitors, and gametes) and preimplantation-stage embryos, including fertilized eggs (zygotes), has been extensively used to obtain genetically engineered animals and is referred to as “germline editing” [5]. As mentioned previously, genome editing of zygotes has now been extensively performed through pronuclear microinjection of genome editing reagents, EP in the presence of genome editing, and direct in situ genome editing of zygotes present in the oviductal lumen of a pregnant female, which is called “GONAD or *i*-GONAD” [3,4]. On the other hand, genome editing of adult organs/tissues has been achieved for genome modifications in various organs/tissues, using physical interventions (e.g., in vivo EP, tail vein injection (also called “intra-venous (i.v.) injection via tail vein”), and nanoparticle-based non-viral gene delivery or viral vector delivery [18,19,20].

Recently, tremendous advances have been made in genome editing at the adult stage for genome modification in various organs and tissues. For example, post-mitotic neurons have been genome-edited through intracerebral delivery of a recombinant adeno-associated virus (rAAV) carrying CRISPR components [21,22] or nanoparticle delivery of CRISPR [23,24]. Hepatocytes have been genome-edited in vivo through i.v. injection of AD vectors, naked plasmid DNA carrying CRISPR components [25], or i.v. injection of rAAVs carrying genome editing components [26]. Immune cells have been genome-edited in vivo through the i.v. injection of nanoparticles carrying CRISPR reagents targeting neutrophils [27], macrophages [28], or T cells [29]. Cancer cells are also targets of CRISPR-based genome editing [30,31,32]. However, therapeutic approaches using the abovementioned genome editing tools in the adult stage often require large amounts of reagents to be administered in vivo, with possible immunological responses against the therapeutic reagents upon repeated administration, and limited ability to deliver them to juvenile cells such as stem cells or actively proliferative cells.

## 3. Various Methods and Routes for In Vivo Gene Delivery in Newborn Pups

To date, there are several methods of gene delivery that target somatic cells and organs. As per the viral vectors, AAV, retrovirus (RV), lentivirus (LV), and adenovirus (AD) are the most widely used vectors for the efficient infection of target organs and cells in vivo [33]. However, they are always associated with the inability to transduce repeatedly, owing to the immunological problems mentioned above. This often results in the integration of viral vectors into host chromosomes, which may cause unexpected abnormalities in individuals.

Among these viral vectors, AAV is now frequently used as a therapeutic reagent for genetic diseases, as well as for other symptoms such as carcinogenesis, because it is usually present as an episomal vector, non-pathogenic, safe, capable of transducing both dividing and non-dividing cells, and rarely integrates into the host genome [34,35]. Furthermore, AAV can transduce neural cells in the post-mitotic stage with high efficiency [36]. Thus, AAV is now a commonly used vector in clinical trials of neurological diseases. This must be balanced with the specific disadvantages associated with AAV vectors: (1) a limited packaging capacity of approximately 4.7 kb [37], and (2) the possibility of a pre-existing humoral response to the capsid protein because of prior wild-type AAV infections [38]. Numerous AAV serotypes have been isolated from humans and nonhuman primates. The first widely used AAV serotype was AAV2, which does not efficiently cross the blood–brain barrier (BBB). Early studies used direct injection of rAAV into the parenchyma or ventricles to target the central nervous system (CNS), resulting in limited CNS distribution and regional transgene expression [39]. Interestingly, AAV9, a serotype newly isolated from human tissues, has been shown to cross the BBB in adult and neonatal animals following i.v. infusion [40,41,42]. Other AAV serotypes (e.g., rAAVrh.8, rAAVrh.l0, rAAVrh.39, and rAAVrh.43) have demonstrated the ability to cross the BBB after i.v. injection of rAAV in adult mice [43,44]. Notably, current rAAVs have strong tropism in the CNS but lack CNS-restricted tropism. For example, rAAV9 confers robust transduction in peripheral tissues such as the liver, heart, lungs, muscles, and pancreas [40]. Recently, Deverman et al. [45] engineered a non-natural AAV capsid, AAV-PHP.B, and demonstrated greater CNS transduction and reduced peripheral transduction compared with AAV9 [44]. AAV genomes predominantly persist in the nucleus as non-replicating episomes [36], and host tissue growth may dilute the transgene and reduce its vector potency during normal aging. This is a problem for the neonatal targeting of organs that consist of cells with high replicative rates, such as the liver, but is less of an issue for the CNS.

Plasmid DNA is also frequently used as a non-viral gene delivery method to transfect tissues or organs of interest [46]. This confers repeated gene delivery owing to the low frequency of eliciting immunological reactivity against DNA. However, their transfection efficiency is generally low compared to that of viral vectors. The delivery of naked plasmid DNA and the peripheral administration of plasmids packaged within nanoparticles have been used for the transfection of skeletal muscle [47,48,49] and neural cells [50,51], respectively, where cell division is not active; therefore, the introduced plasmid can be present in an episomal state for a long period. Notably, the gene expression of naked plasmid DNA introduced into skeletal muscle cells through in vivo EP has been reported to last for at least 1 year [49,52]. However, this method of gene delivery is susceptible to endonuclease degradation. Since then, in vivo EP-based targeted delivery of “naked” plasmid DNA vectors into skeletal muscle myofibers has been optimized [53,54,55]. Hydrodynamics-based gene delivery (HGD) is a novel tail-vein-injection-based method that uses hydrodynamic forces to preferentially transfect hepatic cells [56,57].

To overcome the poor gene transfer efficiency and transient gene expression typically associated with the use of plasmid DNA, Demorest et al. [58] used the Sleeping Beauty (SB) transposable element to achieve chromosomal integration and long-term expression of a luciferase reporter gene and mannitol to increase gene delivery efficiency in newborn pups.

**Figure 1:**
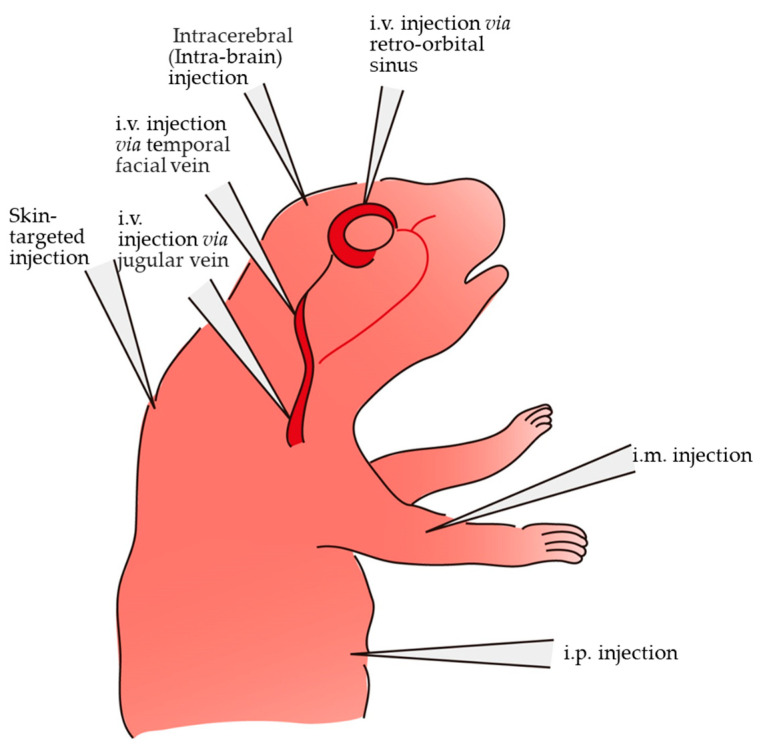
Schematic representation of various routes of neonatal gene delivery: Newborn pups were subjected to intracerebral, intramuscular (i.m.), skin-targeted, or intraperitoneal (i.p.) injection. Solution injection is also possible intravenously (i.v. injection) via the retro-orbital sinus, temporal facial vein, or jugular vein. The figure was drawn in-house.

There are several routes of gene delivery to newborn pups. For example, i.v. injection via the facial vein (Figure 1) is a useful approach for delivering nucleic acids to juvenile pups. This vein (also called the “superficial temporal facial vein”) is localized anterior to the ear bud and is markedly visible on days 2–3 postnatally. However, this method is age-dependent because the facial vein disappears 4 days after birth, and it often requires special skills. To ensure an i.v. injection, a dissecting microscope [59] may be required. Kienstra et al. [60] described additional techniques for intravascular injection in neonatal mice from birth to 6 days of age, termed “i.v. injection via external jugular vein”. Importantly, the use of handheld transillumination is beneficial for the visualization of the vessel, needle position, and injection process, facilitating consistently successful injections. This enhanced visualization extends the use of i.v. injection via the facial or external jugular vein to postnatal day 6 (P6) (on the day of birth, designated as P0.5). Tail vein injection (also called “i.v. injection via tail vein”) is generally employed for gene delivery in adult mice, but it is technically challenging in juvenile mice. According to Prabhakar et al. [59], injection at a young age (≤P21) is essentially impossible, since the tail vein is difficult to visualize or too small to operate on. Retro-orbital sinus injection (Figure 1) is suitable for all murine ages, including newborns and older mice, and is relatively less stressful than tail vein injection. Intracerebral injection (also called “neonatal intra-cranial injection” or “intra-ventricular injection”) of viral vectors, or of naked plasmid DNA and subsequent in vivo EP (Figure 1), is also useful for gene delivery to cells present within a brain (i.e., neural cells, astrocytes, and glial cells). Unlike other organs/tissues, the introduced DNA in the brain is episomally present, and its expression lasts for a long time because it is quiescent for cellular division. Notably, neural stem cells (NSCs) have been reported to exist in the distinct walls of the postnatal lateral ventricle from which radial glial cells (RGCs) arise, and they generate granules and periglomerular (PG) cells during postnatal olfactory bulb (OB) neurogenesis [61]. Targeted gene delivery to defined lateral ventricular walls is currently being explored to study fate specification during postnatal forebrain neurogenesis. Intramuscular (i.m.) injection of nucleic acids (Figure 1) is also considered to be a useful method for enabling long-term expression of introduced genes, because muscular cells do not divide. For example, Kessler et al. [62] demonstrated that after a single i.m. administration of an rAAV vector to adult BALB/c mice, protein (lacZ) expression was detected in myofibers for at least 32 weeks.

Intraperitoneal (i.p.) injection is a useful method for in vivo gene silencing in dorsal root ganglia (DRG) [63,64] (Figure 1). Juvenile skin-targeted gene delivery is considered to be a tool for the stable transfection of skin fibroblasts [65] (Figure 1).

To date, many reports regarding gene delivery to newborn pups have been published using various nucleic acids, such as viral vectors (i.e., AAV, AD, LV, and RV) and non-viral vectors (i.e., naked plasmid DNA and transposon DNA), with the aid of gene-delivery-enhancing tools or approaches such as electroporators and HGD. The past and recent reports on gene delivery in newborn mice are summarized in Table 1. Here, we highlight several interesting methods of neonatal gene delivery.

### 3.1. Correction of Genetic Disorders through i.v. Introduction of Therapeutic Viral Vectors

Genetically inherited disease model mice are considered to be ideal experimental animals for checking the feasibility of neonatal gene therapy, because the consequences of this approach can be readily recognized by the appearance of rescued phenotypes, and those consequences can be directly applied to translational medicine for gene-based therapy in human genetic diseases. To date, numerous gene therapy trials have been conducted using neonates and viral vectors such as AAV- [66,70,77], RV- [67,68], LV- [71,95], and AD-based vectors [69], as well as non-viral vectors [58], via the facial veins of neonatal mice.

Among these, successful gene therapy in neonatal mouse models of mucopolysaccharidosis type I (MPS I) and mucopolysaccharidosis type VII (MPS VII), along with some large animal models, has been demonstrated using RV- [67,68], AD- [69], LV- [71], and AAV-based vectors [66,70,106]. MPS I is one of the most frequent types of genetically inherited lysosomal glycosaminoglycan (GAG) storage disorders, caused by a deficiency in the enzyme α-l-iduronidase (IDUA). Severe Hurler syndrome (severe) presents in infancy with progressive hepatosplenomegaly, upper-airway obstruction, heart disorders, joint stiffness, skeletal dysplasia, corneal clouding, and neurological degeneration. Death can occur during the first decade of life. MPS VII is a lysosomal storage disease caused by the lack of β-glucuronidase (GUSB) activity [108]. This defect results in the progressive accumulation of undegraded GAGs in the lysosomes, leading to lysosomal distention in multiple tissues. The clinical features of MPS VII include skeletal deformities, shortened lifespan, hearing and vision defects, and mental retardation. Thus, neonatal MPS I and MPS VII are appropriate targets for neonatal gene therapy.

Daly et al. [66] employed a neonatal gene therapy approach using a murine model of MPS VII [109] as the initial step in curing lysosomal storage disease. They performed a single i.v. injection with 100 μL of viral suspension containing 5.4 × 10^6^ infectious units (5 × 10^9^ infectious units per kg) of rAAV encoding the human *GUSB* cDNA, via the facial veins of newborn pups (P2). GUSB expression was observed in various tested organs, including the liver, heart, lungs, spleen, kidneys, brain, and retinas, at all timepoints throughout the first 16 weeks of life. Persistent GUSB expression was also noted in multiple areas (including the central gray matter, meninges, cortex, and vessels) of the CNS in rAAV-treated mice at two weeks of age, suggesting that the vectors may pass more readily from the bloodstream into the brain parenchyma of newborns across the BBB. According to Mallard et al. [110], the BBB is present in the neonatal stage; however, several systems, including the transport system and the formation of tight junctions, have not been fully established. In this context, the delivery of therapeutic drugs such as therapeutic nucleic acids to CNS cells at the newborn stage may be possible. This treatment leads to the widespread correction of pathologies, such as lysosomal storage diseases, as exemplified by Gaucher and Pompe diseases. Hartung et al. [70] treated MPS I mice at birth with an rAAV vector carrying human *IDUA* cDNA and achieved excellent biochemical, molecular, and clinical responses, with the correction of multiple parameters of the disease, including metabolic, craniofacial, and neurological abnormalities. Kobayashi et al. [71] performed similar experiments to those of Hartung et al. [70] through facial vein injection of an LV vector (conferring expression of both *Idua* and enhanced green fluorescent protein (EGFP)) using newborn (P1) MPS I model mice (lacking *Idua* expression) [111]. A single injection of LV (1.65 × 10^11^ TU/kg) resulted in the expression of IDUA activity in multiple organs (including the liver, kidneys, spleen, heart, and brain), decreased GAG storage, prevention of skeletal abnormalities, a more normal gross appearance, and improved survival, with the mice being euthanatized 20 weeks after LV injection. They observed that neurons, not astroglia, were the predominant cells transduced into the CNS. The reason for the preferential transduction of neurons over glial cells by the vector remains unknown, but it may be due to differences in local factors such as vascular access and the extracellular matrix or differences in cellular factors that influence susceptibility to transduction.

Hemophilia A (HA) is caused by a deficiency in coagulation factor 8 (F8) and predisposes patients to spontaneous bleeding that can be life-threatening or lead to chronic disabilities. Hu et al. [75] injected a helper-dependent AD vector expressing human F8 via the ciliary veins of neonatal HA KO mice. Three days later, the mice produced high levels of F8. These levels declined rapidly with the animals’ growth until 5 weeks of age. Subsequent re-administration and augmentation of expression were possible, as operational tolerance was established for F8. The same group [77] next investigated the use of rAAVrh10 expressing human F8 to treat this severe phenotype via gene delivery at the neonatal stage. They demonstrated that the i.v. injection of rAAVrh10 via the facial veins of neonatal mice with the HA phenotype (P2) resulted in long-term correction and avoidance of immune responses.

### 3.2. Tissue Tropism among rAAV Serotypes as Revealed by i.v. Introduction of rAAVs to Neonates

As mentioned previously, there are several serotypes, each of which displays different cell-type specificity upon transduction. For example, Pacak et al. [73] tested the ability of rAAV2/1, rAAV2/8, and rAAV2/9 (1 × 10^11^ vector genomes (vg)) to show preferential cardiac transduction through i.v. injection of rAAVs (35 μL; carrying *lacZ* gene) via the facial veins of neonatal mice (P1). These recombinants or pseudotyped vectors were created by inserting a transgene of interest flanked by inverted terminal repeats (ITRs) of AAV2 into the capsid of another serotype. At 4 weeks post-injection, both rAAV2/8 and rAAV2/9 were able to transduce tissues such as the brain, lungs, and kidneys, but rAAV2/9 displayed the highest natural affinity for the heart. Similar experiments were performed by Hammond et al. [98], who tested whether rAAV2/1, rAAVDJ8, and rAAV9 exhibited tropism in different brain regions and cell types. Each rAAV was administered to neonatal mice (P0) via intracerebroventricular injection (ICV). The brains were systematically analyzed for GFP expression at 3 or 6 weeks post-infection in various regions, including the OB, striatum, cortex, hippocampus, substantia nigra (SN), and cerebellum. The rAAV2/1 infections were more prevalent in the cortical layers but penetrated the midbrain less frequently than rAAVDJ8 and rAAV9. Additionally, differences were observed in the persistence of viral transgene expression among the three serotypes examined in vivo. For example, rAAVDJ8 displayed more tropism in astrocytes than rAAV9 in the SN region. Hammond et al. [98] suggested that specific AAV serotypes are required to selectively deliver the transgenes of interest to different brain regions in both astrocytes and neurons.

The potential of rAAV-mediated inner-ear transgene delivery in early postnatal mice was also assessed by Shibata et al. [83], who administered an i.v. injection of rAAV2/9 carrying an *EGFP* reporter gene into wild-type neonatal mice (P0–1). When these treated mice were analyzed three weeks later, binaural transduction of inner hair cells, spiral ganglion neurons, and vestibular hair cells was observed. The transduction efficiency increased in a dose-dependent manner. The inner hair cells were transduced in an apex-to-base gradient, with transduction reaching 96% at the apical turn. Hearing acuity in the treated animals was unaltered on postnatal day 30. Transduction is influenced by viral serotype and age at injection, with less efficient cochlear transduction observed with the systemic delivery of rAAV2/1 and in juvenile mice with rAAV2/9. Collectively, these data validate i.v. delivery of rAAV2/9 as a novel and atraumatic technique for inner-ear transgene delivery in early postnatal mice. This type of gene therapy is a promising approach for preventing hearing loss or restoring hearing after the loss has occurred.

### 3.3. Gene Delivery to Peripheral Tissues through i.v. Introduction of rAAVs in Neonates

Lewy bodies, a pathological hallmark of Parkinson’s disease, are routinely identified in neurons of the enteric nervous system (ENS) following colon biopsies. The ENS is the intrinsic nervous system of the gut and is responsible for coordinating the secretory and motor functions of the gastrointestinal tract. Notably, ENS transduction following systemic vector administration has not been thoroughly evaluated. Gombash et al. [80] showed that systemic injection of rAAV2/9 (carrying *GFP* cDNA) into neonatal (P1) or juvenile (P21) mice resulted in the transduction of 25–57% of ENS myenteric neurons. Transgene expression was prominent in choline-acetyltransferase-positive cells, but not in vasoactive intestinal peptide or neuronal nitric oxide synthase cells, suggesting a bias for cells involved in excitatory signaling. Transduction of rAAV2/9 in enteric glia was very low compared to that in CNS astrocytes. Enteric glial transduction was enhanced using a glia-specific promoter. Furthermore, the authors showed that rAAV2/8 resulted in transduction in neonatal mice comparable to that of rAAV2/9, although rAAV2/1, 2/5, and 2/6 were less efficient. These data demonstrate that systemic rAAV2/9 has a high affinity for peripheral neural tissue and is useful for future therapeutic development and basic studies on the ENS.

Karda et al. [6] generated somatic Tg rodents to monitor signaling pathways in organs using whole-body bioluminescence imaging, through neonatal i.v. delivery of rAAV8 carrying *EGFP* cDNA. They observed widespread gene expression in the central and peripheral nervous system, liver, kidneys, and skeletal muscles. Next, they performed an i.v. injection of rAAV8 containing the firefly luciferase gene and *EGFP* cDNA under transcriptional control of a constitutive spleen focus-forming virus (SFFV) promoter and a nuclear factor-κB (NF-κB) binding sequence for biosensor evaluation of lipopolysaccharide (LPS)-induced inflammation. A single dose of LPS caused 10-fold increased luciferase expression in AAV8-NFκB mice and EGFP expression in astrocytes and neuronal cells. Importantly, whole-body bioluminescence persisted for up to 8 months. According to Karda et al. [6], this technology can complement existing germline Tg models, and it may be applicable to other rodent disease models.

### 3.4. Neonatal i.m. Injection

Exogenous gene transfer into skeletal muscle fibers has been developed in two forms: (1) viral-mediated delivery using rAAV vectors [112,113], and (2) non-viral delivery of “naked” plasmid DNA vectors [23,24] for the purpose of secreting the therapeutic proteins (produced from the injected transgene) into bloodstream for relatively long periods. This is also true for neonatal i.m. injection (Figure 1). For example, Daly et al. [106] demonstrated that neonatal i.m. injection of rAAV resulted in prolonged *GUSB* cDNA expression in situ and the correction of liver pathology in MPS VII model mice. Notably, at the neonatal stage, up to 7 days after birth, mice exhibit immunological tolerance against the exogenous substances administered [114], which means that repeated administrations are theoretically possible when naked DNA is administered early, because no immune response should be generated against the exogenous transgenes. Signori et al. [107] tested this possibility through the i.m. injection of a plasmid DNA (20 μg) encoding human apolipoprotein E3 (*APOE3*) cDNA in newborn mice (showing a hypercholesterolemic phenotype, due to genetic disruption of the endogenous *ApoE* gene) at the ages of 5 (a stage showing immunological tolerance) and 14 (a stage showing immunological intolerance) days. They showed that direct i.m. injection of naked DNA reduced severe hypercholesterolemia in newborn mice. Moreover, when naked DNA is administered early, no immune response is generated against human APOE, allowing for repeated administration. However, repeated i.m. injection at day 14 resulted in the production of antibodies against the injected plasmids. Based on these findings, Signori et al. [107] proposed a concept called “neonatal therapy”, which is important for the treatment of many genetic childhood diseases where early and repeated administration is required to prevent long-term damage arising from abnormal genetic conditions.

### 3.5. Neonatal i.p. Injection

RNA interference (RNAi) using short interfering RNAs (siRNAs) or short hairpin RNAs (shRNAs) in neurons has been used to study gene functions and identify new targets for disease intervention. Almost all data were obtained from experiments using germline transgenesis. In contrast, in vivo delivery of RNAi through i.p. administration in neonates (Figure 1) has proven useful for the suppression of GOIs, especially in the lower motor neurons (LMNs) and DRG. For example, Foust et al. [74] attempted to target LMNs using rAAV8, which showed a dispersed transduction pattern after i.v. or i.p. injection into neonatal mice via entry into the nervous system. Gene delivery to LMNs can be useful for disorders such as spinal muscular atrophy and amyotrophic lateral sclerosis, because LMNs reside in the ventral gray matter of the spinal cord and send axonal projections to innervate the skeletal muscle. When i.p. injection with rAAV8 carrying the *GFP* gene was performed in neonatal mice (P1), the spinal cords were positive for rAAV genomes at both 5 and 14 days post-injection. Notably, extensive labeling of the fibers was observed in the dorsal horns and columns, indicating dorsal root ganglion transduction across all timepoints and injection routes. These findings suggest that systemic injection of rAAV8 is not an effective delivery route to target LMNs but could be useful for targeting sensory pathways in chronic pain.

Machida et al. [64] developed a highly efficient method for in vivo gene silencing in the DRG using shRNA-expressing rAAV9. Intraperitoneal (i.p.) administration (200 μL) of single-stranded (ss) rAAV9 (ssAAV9-shRNA) (1.0 × 10^13^ vg/mL) to neonatal mice (P1) resulted in an approximately 80% reduction in target (superoxide dismutase 1 (*Sod1*)) mRNA in the DRG, as well as 74.7% suppression of the protein. There were no major side effects, and the suppressive effect lasted for more than three months after the injection of rAAV9. Machida et al. [64] concluded that i.p.-injection-mediated delivery of rAAV carrying shRNA would be useful for creating animal models to investigate the molecular mechanisms of pain and sensory ganglionopathies.

### 3.6. Gene Delivery via the Retro-Orbital Sinus or toward Retinal Cells

The eye is an ideal organ for therapeutic gene treatment because it is immune-privileged and easily accessible, and direct viral delivery primarily results in local infections. Since the eye is not a vital organ, mutations in eye-specific genes tend to be more common. To date, more than 40 eye-specific genes harboring blindness-related mutations have been identified [115]. In addition to the in vivo transfection of retinal cells, gene delivery through the retro-orbital sinus is an effective alternative to tail-vein-mediated injection, which is especially useful when tail veins are difficult to visualize or too small to operate on.

#### 3.6.1. Retinal Gene Delivery into the Subretinal Space

**Figure 2:**
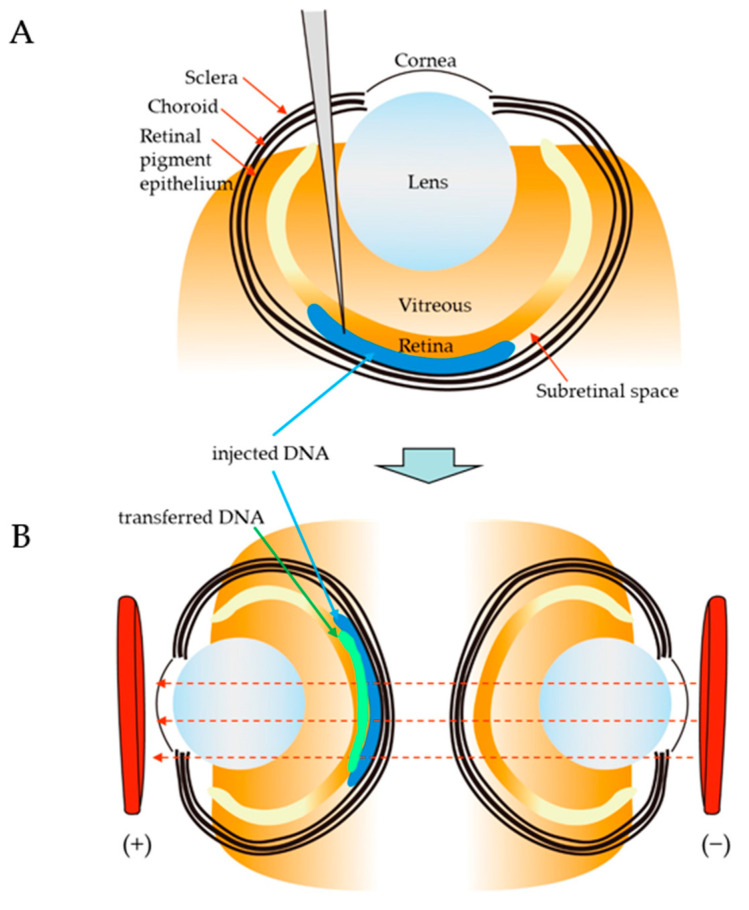
Schematic of in vivo gene delivery via subretinal injection into newborn mouse pups: (**A**) Subretinal injection. The blunt Hamilton needle was pushed through the retina as described by Venkatesh et al. [115]. The needle stops at the sclera if it is not pushed hard, because of the tougher composition of the sclera. (**B**) In vivo electroporation (EP) at the nucleic acid injection site. After the injection into the central region of the retina, the tweezer electrodes were positioned correctly and electroporated. The injected DNA was transferred to the retina along with an electric field generated by the electroporator. Figures were drawn in-house and reproduced as described by Venkatesh et al. [115].

Matsuda and Cepko [102] first demonstrated that the rodent retina could be successfully transfected after the injection of plasmid DNA into the subretinal space of a newborn pup and subsequent in vivo EP towards the entire head (Figure 2). Newborn mouse pups were anesthetized by chilling on ice, and a small incision was made in the eyelid and sclera near the lens using a 30-gauge needle. A solution (~0.5 μL) containing plasmid DNA (3~6 μg/μL) and 0.1% Fast Green in phosphate-buffered saline (PBS) was injected into the subretinal space through the incision using a Hamilton syringe with a 32- or 33-gauge blunt-ended needle under a dissecting microscope. After DNA injection, tweezer-type electrodes (7 mm diameter) briefly soaked in PBS were placed to softly hold the heads of the pups, and five square pulses (80 V with a 50 ms duration at 950 ms intervals) were applied using a pulse generator. Using this technique, GFP expression vectors were efficiently transfected into retinal cells, with minimal damage to the operated pups. The transfected GFP allowed for clear visualization of cell morphology, and expression persisted for at least 50 days. DNA-based RNAi vectors directed against two transcription factors that are important for photoreceptor development led to photoreceptor phenotypes similar to those of the corresponding KO mice. Reporter constructs carrying retinal-cell-type-specific promoters were introduced into the retina in vivo, where they exhibited appropriate expression patterns. This gene delivery approach, targeting the retina, will be useful for the treatment of retinal diseases such as retinal dystrophies.

As mentioned above, in vivo EP after retina-targeted gene delivery has proven useful for introducing GOIs into neonatal rodents. Matsuda and Cepko [103] attempted to perform temporal and spatial regulation of the expression of introduced genes using a Cre/*loxP*-mediated inducible expression system (for the temporal regulation of GOI) and cell-type-specific promoters (for the spatial regulation of GOI). For example, Matsuda and Cepko [103] injected plasmid DNA into the subretinal space of a newborn pup (P0) and subsequently performed in vivo EP on the entire head, using the method described in their previous paper [102]. They used the plasmid CAG-CreERT2, in which the expression of a conditionally active form of the Cre recombinase CreERT2 is controlled by the ubiquitous CAG promoter [116]. ERT2 has a mutated ligand-binding domain of the estrogen receptor (ER). Cre recombinase is activated in response to 4-hydroxytamoxifen (4OHT). With Cre recombinase, a Cre-dependent expression vector CALNL-DsRed that contains the CAG promoter, a floxed “stop cassette” and a reporter gene (DsRed) were used as recombination indicators. When P0 rat retinas were co-electroporated with CAG-CreERT2, CALNL-DsRed, or CAG-GFP (transfection control) and harvested at P21, very high background recombination (DsRed expression) was detected without 4OHT. When 4OHT was i.p. injected into transfected rats at P20, the induction of DsRed expression was clearly detected 24 h after 4OHT administration. These results indicate that combinations of these constructs facilitate various experiments, including cell-type-specific gene expression, conditional RNAi, and fate mapping of progenitor and precursor cells.

#### 3.6.2. Gene Delivery via the Retro-Orbital Sinus

HGD hepatocytes are the most effectively transfected [56,57]. Unfortunately, HGD is often technically challenging when used in animals whose tail veins are difficult to visualize or are too small to operate on. To overcome this limitation, Yan et al. [104] developed an alternative in vivo gene delivery method through rapid injection (within approximately 8 s) of a large volume of solution (i.e., 10 µg of plasmid DNA carrying the luciferase gene, diluted to 10% of the body weight in normal saline) through the retro-orbital sinus of neonatal mice (P0). Efficient expression of exogenous genes was specifically detected in the livers of the treated animals, as observed in HGD-treated adult mice [56,57]. This approach will be beneficial for exploring gene functions in hepatic cells, and for treating genetic diseases.

Numerous gene therapy trials have been conducted using neonates and viral vectors, such as AAV [77,78], RV [101], LV [5], and AD [105], via the retro-orbital sinus of neonatal mice. For example, VandenDriessche et al. [101] demonstrated that i.v. injection of RV (100 μL) carrying a B-domain-deleted *F8* cDNA via the retro-orbital sinus of neonatal mice with the HA phenotype (P2–P3), using a 30-gauge needle and a 1 mL syringe, resulted in long-term correction and correction of HA in those mice. Dalkara et al. [78] used a “double tyrosine mutant of AAV9” to transduce the retina with high efficiency via neonatal intravascular administration at P1. The double-tyrosine mutant of AAV9, i.e., rAAV9, has tyrosine-to-phenylalanine mutations at two highly conserved sites and has been previously shown to increase the infectivity of several AAV vectors [117]. In adult mice, successful gene delivery was discernible in the CNS and retina. Particularly, rod photoreceptor cell-specific gene expression was detected when a rhodopsin promoter was incorporated into rAAV9. This approach may be useful for developing retina-targeted gene therapies, or for creating animal models of neurodegenerative diseases. Notably, Bemelmans and colleagues [79] obtained results similar to those of Dalkara et al. [78] through the i.v. injection of an rAAV9 vector encoding GFP, which could direct efficient cell transduction in the adult CNS. Transduction of all layers of the retina, including retinal pigment epithelium cells, photoreceptors, bipolar cells, Müller cells, and retinal ganglion cells, was confirmed in adult mice despite the presence of a mature blood–eye barrier. The cells on the inner side of the retina, including ganglion cells, were transduced with the highest efficiency. Bemelmans et al. [79] concluded that this intravascular gene transfer approach is beneficial for eliminating the potential invasiveness of ocular surgery and can be used as a new gene therapy for retinal diseases.

### 3.7. Intracerebral Injection

Developing a system for widespread somatic gene transfer in the CNS is beneficial for understanding the global influence of exogenous genes in animal models and performing functional gene analysis in vivo. This can be achieved through intracerebral injection (also called “neonatal intra-cranial injection,” “ICV”, “intra-thecal (IT) injection”, or “intra-ventricular injection”) at the neonatal stage (Figure 1).

Passini and Wolfe [86] first injected 2 µL of rAAV2 vector into each cerebral lateral ventricle of pups (P0.5; anesthetized on ice) with a finely drawn glass micropipette needle to map its distribution and transduction pattern. The viral solution contained 0.05% (wt/vol) trypan blue to determine whether the ventricles had been injected. Only pups in which the lateral ventricles were filled with the viral solution were analyzed. Structure-specific patterns of expression were observed in some of the major principal neuron layers, and these patterns were maintained for at least one year. Notably, the transduction patterns were explained by the circulation of the viral vector in the subarachnoid space via cerebrospinal fluid flow, followed by the transduction of underlying structures, rather than by progenitor cell infection and subsequent migration. The authors concluded that gene transfer throughout the CNS can be achieved without germline transgenesis, and they established an experimental strategy for introducing genes into somatic cells in a highly predictable manner.

A similar approach has been reported for other AAV serotypes [94,97,98]. For example, Kim et al. [97] demonstrated that intraventricular injection of rAAV at the neonatal stage (P0–P1) led to widespread viral transduction of neural subsets spanning every region of the brain, from the olfactory bulbs to the brain stem. Similar observations were reported by Kim et al. [93] and Chakrabarty et al. [94]. The virally delivered transgenes persisted for up to one year after transduction. This versatile manipulation technique has enabled studies on topics ranging from early postnatal brain development to aging and degeneration in adults.

**Figure 3:**
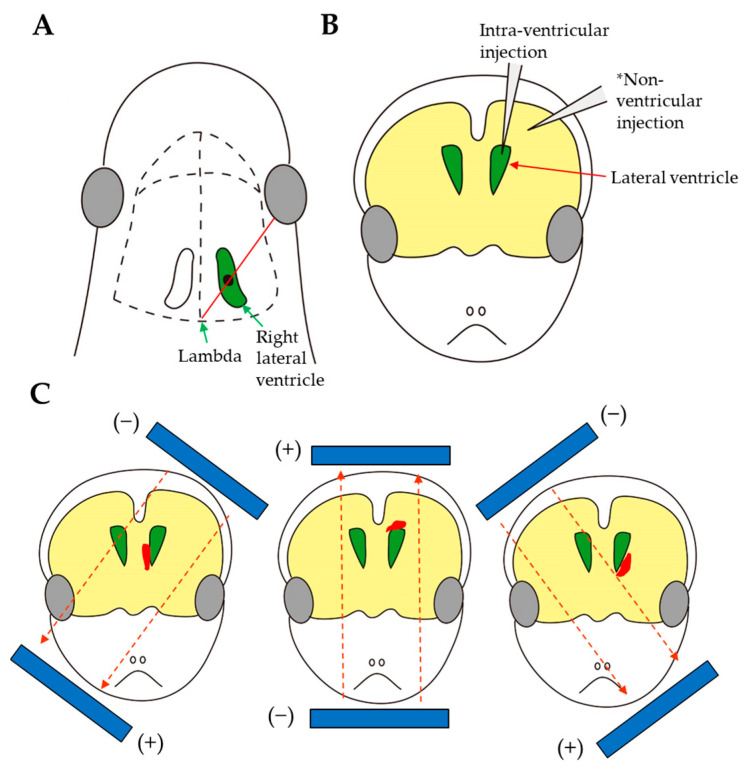
Gene delivery to the postnatal forebrain via intraventricular injection of DNA and subsequent in vivo electroporation (EP): (**A**) Location of the point through which the intracerebral DNA injection was performed. According to Boutin et al. [88], following anesthesia with hypothermia, a pup at P0 was fixed on a custom-made support plate. A virtual line (red) connecting the right eye to the craniometric landmark lambda (visualized using a strong cold light source) was used, and the incision (indicated by a dot) was positioned 1 mm caudal to the midpoint of this line as a positional marker for DNA injection. (**B**) Intracerebral injection of nucleic acids. For intraventricular injection, a hole was made in the skull with a 31-gauge needle (without exposing the skull), and a micropipette containing nucleic acids and dye was inserted perpendicular to the surface of the skull at a depth of 2.5 mm from the skull surface into the lumen of the right lateral ventricle, and the plasmid solution (1–1.5 μL) was injected. An injection was considered correct when the dye spread throughout the lateral and third ventricles and was visible under a light source. For non-ventricular injection, a micropipette was inserted at a depth of approximately 1 mm from the skull surface into the parenchyma of the brain (indicated by *). (**C**) Differences in in vivo EP efficiency using different electrode locations based on study by Fernadez et al. [92]. A schematic representation of the electrode locations is also presented. The red area near the lateral ventricle indicates successful transfection of the putative area. These figures were drawn in-house and reproduced based on the works of Boutin et al. [88] and Fernadez et al. [92].

In addition to the use of viral vectors, a method for the intracerebral introduction of non-viral vectors, such as plasmid DNA, and subsequent in vivo EP into the entire brain is known. For example, Boutin et al. [88] presented a new method for the efficient introduction of nucleic acids into the postnatal mouse forebrain (P0–P4) through intraventricular injection of plasmid DNA (carrying *EGFP* cDNA; 5 μg/μL), followed by in vivo EP (~100 V with five electrical pulses (50 ms, separated by 950 ms intervals) using the CUY21 edit device (Nepagene, Chiba, Japan) and 10 mm tweezer-type electrodes coated with conductive gel) (Figure 3A,B). The depth of capillary injection into the lumen of the right ventricle was between 2.5 mm (P0) to 3.5 mm (P4). Two days after intraventricular injection, strong expression of the transgenes was detected in the radial glia, neuronal precursors, and neurons of the olfactory system. Interestingly, the injection and EP processes had no obvious consequences on the behavior of the surviving pups. After warming, the animals began suckling immediately and were indistinguishable from their non-manipulated littermates within 15 min. Boutin et al. [88] performed two proof-of-principle experiments to demonstrate the usefulness of this approach for functional gene analysis. First, the expression of neural cell adhesion molecule (NCAM; hNCAM-140) in NSCs strongly interfered with the maintenance of radial glia and induced the generation of migratory neuronal precursors. Second, the overexpression of the cell-cycle inhibitor p21 interfered with the proliferation of NSCs. The authors concluded that this approach is an important tool for future studies on postnatal neurogenesis and neural development.

Postnatal OB neurogenesis involves generation of granules and PG cells by NSCs in the walls of the lateral ventricle. Granules and PG cells are the two main classes of OB-inhibitory interneurons that are generated after birth from stem cells located in the subventricular zone (SVZ). However, the mechanisms by which new neurons are generated, how they integrate into circuits, and their roles in coding remain unclear. To assess this issue, neonatal intracerebral injection of plasmid DNA and subsequent in vivo EP were performed by Chesler et al. [89], who injected 1–2 µL of plasmid (1–5 µg/µL) into the lateral ventricles of mice (P0–P4) along the rostral migratory stream to the OB, followed by 5 pulses of 150 V with a 50 ms duration, at 950 ms intervals, delivered with tweezer-type electrodes. This study aimed to examine whether neuroprogenitors (NSCs) in the SVZ give rise to neuroblasts that migrate along the rostral migratory stream (RMS). This EP approach results in the widespread expression of foreign genes in specific cell populations, indicating that EP can label diverse progenitor populations within the SVZ. Importantly, less than 0.5% of GFP+ cells were labeled with activated caspase-3 antibodies or TUNEL, indicating that EP does not accelerate cell mortality. Additionally, neurons labeled via EP were electrophysiologically indistinguishable from neurons derived from non-electroporated animals. Chesler et al. [89] also demonstrated the usefulness of genetically encoded sensors as a noninvasive tool for studying neuronal function in vivo, as well as the EP-based introduction of multiple genes to express them simultaneously in a single cell. This EP-based gene delivery approach targeting lateral ventricle NSC populations is valuable for studying the genetic factors involved in forebrain neuronal specification. Similar to the results of Chesler et al. [89], Fernandez et al. [92] refined the EP of the postnatal forebrain as a technique for the precise and accurate delivery of transgenes to NSCs located in distinct walls of the lateral ventricle in mice (Figure 3C). Consequently, NSCs in the distinct walls of the lateral ventricles produce neurons that invade different layers of the OB. Ito et al. [96] established a stable gene transfer method to analyze the RMS and postnatal OB neurogenesis in vivo through EP following gene delivery (1 µL of plasmid DNA (0.5 µg/µL) + 0.2% Fast Green) to the lateral ventricles of neonatal mouse brains (P0) by piercing through the skin and skull, similar to the previously published methods [88,89,92]. They carefully determined the in vivo EP conditions, such as the position and direction of the electrode, the voltage for electric pulses, and the interval between EP and sample preparation. The correct injection was confirmed by recognizing the darkly stained shape of the lateral ventricle under light. Consequently, GFP+ cells in the dentate gyrus (DG) extended branched dendrites and long axons into the molecular layer and hilus, respectively, 21 days after EP. Interestingly, the expression of GFP in dentate neurons was sustained for at least nine months after in vivo EP. Thus, this method is both time- and cost-effective and is a rather simple technique that facilitate the screening of molecules responsible for postnatal neurogenesis in the DG.s

Molotkov et al. [91] developed a method for transfecting non-migratory cell types into the brain via postnatal non-ventricular microinjection. In more detail, a solution (25 to 100 nL) containing plasmid DNA (carrying *EGFP* cDNA; 1–3 μg/μL) and 0.1% Fast Green was injected. Subsequently, in vivo EP was performed on the injection site at P2, which allowed for the targeted delivery of genes directly to a small region of interest (e.g., deep cortical layers or the striatum region) in the newborn rat brain. This approach is also called “intra-parenchymal administration of nucleic acids”. These animals can be used for two-photon in vivo imaging or electrophysiological experiments on acute brain slices.

### 3.8. Gene Delivery to the Skin Cells of Newborn Mice

To achieve stable transfection of skin cells in vivo, Titomirov et al. [65] performed subcutaneous injection of a solution (60 μL) containing two plasmid DNAs (12 μg each; a pSV3neo plasmid carrying a neomycin-resistance gene, and a pHEB4 plasmid containing the E1A region of AD 2, which could immortalize primary rodent cells) into the skin cells of newborn mice (at P1–3). After an adsorption period (10–15 min), the injected portion was subjected to in vivo EP (400 V/cm^2^ with pulse width of 100 μs) using the laboratory-built special device (two flat stainless steel electrodes of about 2 cm^2^ mounted on a plastic clip). Electric pulses of up to 400 V did not cause visible skin damage. Neomycin-resistant colonies composed of fibroblasts were found in primary cell cultures obtained from the treated skin. In four independent experiments, the transformation efficiency was 0.2–7.0 × 10^−4^. Southern and Northern blotting demonstrated that all six clones tested exhibited chromosomal integration of the transgenes and their expression. These experiments show that in vivo EP is useful for obtaining stably transfected murine skin cells. Juvenile skin-targeted gene delivery appears to have various applications, such as exploration of the mechanisms underlying the multistep carcinogenesis of skin cells, treatment of pathological conditions resulting from the loss or malfunction of a physiologically important protein, or preparative-scale production of heterologous proteins in vivo.

## 4. Genome Editing at Neonatal Stages

Neonatal gene therapy is a promising strategy for treating several congenital diseases that are diagnosed shortly after birth through the systemic or local administration of genes coding for therapeutic proteins. However, their expression must be maintained during postnatal life to prevent potential pathological consequences. Subsequent re-administration and augmentation of expression for this purpose are theoretically possible, but tremendous effort is required to achieve this goal. Genome editing is a powerful technique for inducing mutations or correcting a target gene through a single step in the gene delivery approach. Once this event occurs, gene delivery is not required. To date, there have been few reports on genome-editing-based gene therapy, and all of these were experiments using rAAV carrying CRISPR/Cas9 components and animal models for human genetic disorders, such as blood clotting and liver disorders (Table 2). In this section, we present the typical cases of successful gene correction for genetic disorders.

### 4.1. Neonatal Gene Correction in Genetic Liver Disorders

Many genetic liver diseases that appear in the pediatric or juvenile stages are lethal. Organ transplantation remains the only curative treatment for metabolic crises. Gene therapy using non-integrating viruses such as rAAV has shown efficacy in adult patients. However, treatment during the early stages of development may not be optimal, because of the rapid loss of episomal viral DNA as developing hepatocytes proliferate. However, HDR-based gene correction of a target gene at the newborn stage using the CRISPR/Cas9 system appears to be a promising approach for treating genetic liver diseases, because this type of gene correction does not require chromosomal integration of transgenes and is completed within a short period (probably within a day) after transduction.

According to Yang et al. [118], an X-linked deficiency in the urea cycle disorder enzyme ornithine transcarbamylase (OTC) in humans causes recurrent and life-threatening episodes of hyperammonemia. In males that are hemizygous for *OTC*, the first metabolic crisis usually occurs during the newborn period and is associated with up to 50% mortality. In an animal model of *OTC* deficiency, male sparse-fur-ash (spf^ash^) mice had a G-to-A point mutation at the donor splice site at the end of exon 4 of the *OTC* gene, which led to abnormal splicing and a 20-fold reduction in *OTC* mRNA and protein levels. Affected animals had 5% residual OTC activity and could survive on a chow diet; however, they developed hyperammonemia, which can be lethal when fed a high-protein diet.

**Figure 4:**
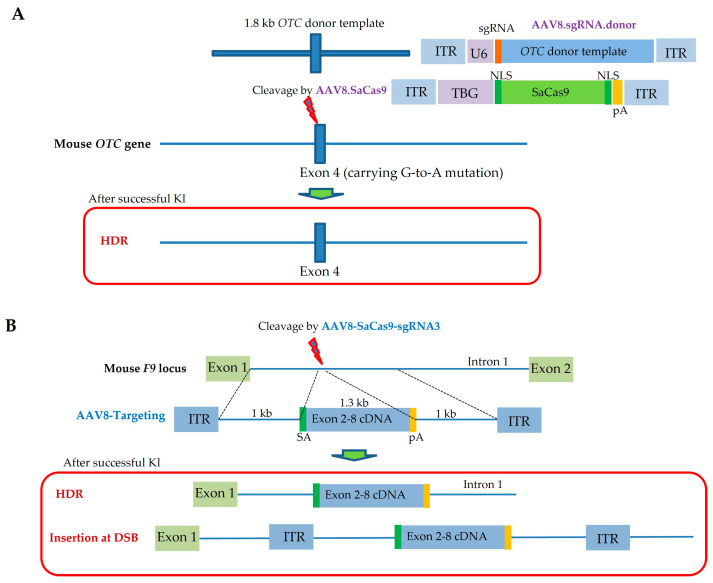
Schematic representation of an rAAV-mediated KI strategy: (**A**) HDR-based KI using dual rAAV vectors based on data from Yang et al. [118]. The spfash mutation (G-to-A mutation) in the spfash mouse is located in exon 4 of the *OTC* locus. The sequence recognized by single-guide (sg) RNA is present around the mutation site. The AAV8.sgRNA.donor vector contains a 1.8 kb normal murine OTC donor template sequence covering the mutation site and sgRNA expression unit (driven by the U6 promoter). The AAV8 SaCas9 vector contains a liver-specific *TBG* promoter and the *SaCas9* gene. (**B**) HDR- or insertion-mediated KI using dual rAAV vectors, based on the work of Ohmori et al. [119]. AAV8-SaCas9-sgRNA3 induces a DSB in intron 1 of the murine *F9* gene. AAV8-targeting is a vector that induces gene correction via HDR or insertion into the DSB. When these two vectors are introduced to target cells, HDR-mediated KI of normal cDNA comprised by exons 2–8 of the *F9* gene, which is designated as “HDR”, occurs. On the other hand, NHEJ-mediated KI of the target sequence (normal cDNA), which is designated as “Insertion at DSB”, also occurs. ITR, inverted terminal repeat; NLS, nuclear location signal; pA, poly(A) site; SA, human *F9* intron 1 splice acceptor site; *F9* exons 2–8, codon-optimized cDNA (exons 2–8) from mouse *F9*. These figures were drawn in-house and reproduced based on the works of Yang et al. [118] and Ohmori et al. [119].

Yang et al. [118] first demonstrated that HDR-based correction of metabolic liver disease is possible when two types of rAAV8—one with *Staphylococcus aureus*-derived Cas9 (*SaCas9*) and a liver-specific thyroxine-binding globulin (*TBG*) promoter, and the other with gRNA/donor DNA—are co-injected intravenously into newborn mice (P2) with a partial deficiency in *OTC* (Figure 4A). In this study, 5 × 10^11^ genome copies (GCs) of rAAV carrying a gRNA/donor and 5 × 10^10^ GCs of rAAV carrying SaCas9 were used for all newborn mouse experiments. Analyses performed at 1, 3, and 8 weeks after rAAV administration demonstrated that the mutation in 10% (6.7–20.1%) of hepatocytes was successfully reversed, which was also accompanied by increased survival in mice challenged with a high-protein diet, which exacerbates disease. Notably, gene correction in adult *OTC*-deficient mice was lower and was accompanied by larger deletions that ablated the residual expression of the endogenous *OTC* gene, leading to diminished protein tolerance and lethal hyperammonemia on a chow diet. This event (remarkably high frequency of indels) was observed at both low and high vector doses (1 × 10^11^ vs. 1 × 10^12^ GCs for SaCas9-expressing rAAV8; 1 × 10^12^ vs. 5 × 10^12^ GCs for rAAV8 carrying gRNA and donor DNA), with efficiencies ranging from 45% to 42%, respectively. In contrast, the reversion rates of G-to-A mutations were 0.3% and 1.7%, respectively. In adult spf^ash^ mice, higher and/or more persistent SaCas9 expression may have caused these indels. However, the quantification of *SaCas9* mRNA revealed that *SaCas9* mRNA levels were lower in 3-week adults than in any liver tissue harvested up to 8 weeks after injection in newborns. Importantly, immunofluorescence analysis of the liver for OTC expression revealed a pattern of isolated OTC+ cells in adults, whereas clusters of OTC+ cells were present in newborns, suggesting that newborn hepatocytes were proliferative, whereas adult hepatocytes were not. Notably, HDR preferentially occurs in dividing cells, whereas NHEJ occurs in both dividing and non-dividing cells [11]. It is likely that the findings of Yang et al. (low efficiency of HDR in adult hepatocytes) [118] reflect the low proliferative ability of adult hepatocytes. Alternatively, as pointed out by Yang et al. [118], different NHEJ mechanisms may exist in non-dividing adult hepatocytes and dividing newborn hepatocytes, affecting the quality of the DNA repair response. HDR-based gene correction in newborns may be more plausible than in adults, since almost all cells (except for neuronal and skeletal cells) in newborn pups are in a state where cells proliferate actively.

### 4.2. Neonatal Gene Correction in Genetic Blood Clotting Disorders

Hemophilia B (HB) is an X-linked recessive bleeding disorder caused by a defect in the gene encoding coagulation factor F9. The current treatment for HB, which entails lifelong injections of F9, is expensive, difficult to adhere to, and non-curative.

Ohmori et al. [119] recently demonstrated the possibility of using CRISPR/Cas9 to rescue abnormal phenotypes caused by HB. They created two types of rAAV8, called “AAV8-SaCas9- single-guide (sg) RNA3” and “AAV8-Targeting” (Figure 4B). The former is a vector comprising *SaCas9* and sgRNA (which induces a DSB in intron 1 of the murine *F9* gene). The latter is a vector comprising exons 2–8 of the *F9* gene and is used as donor DNA to induce gene correction via HDR- or NHEJ-mediated insertion at the DSB. The two rAAV vectors were i.v. injected into neonatal pups (P0) with HB. Notably, silent mutations were incorporated in the sgRNA recognition site of the donor sequence to avoid re-cleavage by SaCas9 after the knock-in (KI) of normal *F9* cDNA. When CRISPR-mediated cleavage and subsequent HDR occur in the infected site, the normal *F9* cDNA is successfully inserted into the DSB site, which is designated as “HDR” (Figure 4B). On the other hand, insertion of the target sequence at the DSB site using NHEJ also occurs, which is designated as “Insertion at DSB” (Figure 4B). According to Ohmori et al. [119], “Insertion at DSB” was more effective at increasing plasma F9 levels compared with “HDR”. Consequently, mutation-specific gene editing in newborns with HB was successful, resulting in increased production of F9 to sufficiently correct the disease phenotype. They suggested that the ongoing hemophilia therapy targeting the antithrombin gene with antisense oligonucleotides could be replaced by the SaCas9/sgRNA-expressing rAAV8 vector.

A similar approach was also used by Wang et al. [120], who performed HDR-based gene correction through neonatal i.v. injection of dual rAAV8 vectors (Cas9-expressing rAAV8 and rAAV8 carrying gRNA/donor DNA containing *F9* cDNA, in which a hyperactive F9 Padua mutation was introduced) into *F9* KO mice and found that expression of F9 at a normal level persisted for over 8 months. Furthermore, the serum F9 level was maintained for 24 weeks, even after partial hepatectomy, suggesting “stable genomic targeting”. Wang et al. [120] concluded that the CRISPR approach is useful for achieving the lifelong expression of therapeutic proteins.

### 4.3. Neonatal Gene Correction in Muscular Dysfunction

Duchenne muscular dystrophy (DMD) is a devastating disease that affects approximately 1 in 5000 male births and is caused by mutations in the dystrophin gene. Genome editing can restore the expression of a modified dystrophin gene at its native locus to modulate disease progression.

Recently, three groups [123,124,125] simultaneously reported phenotypic corrections using CRISPR/Cas9 in a mouse model (*mdx*) of DMD after birth. They induced DSB on both sides of an abnormal exon using two rAAV vectors to promote permanent exon skipping. For example, Nelson et al. [124] used dual rAAV8 (one with *SaCas9* cDNA and the other with two gRNA expression cassettes (targeted to introns 22 and 23)) to remove mutated exon 23 from the dystrophin gene in *mdx* mice. It is expected that simultaneous DNA cleavage in both introns by Cas9/gRNA complexes would remove exon 23 from the genome, resulting in the production of an internally truncated but highly functional dystrophin protein in the skeletal myofibers and cardiac muscle. The rAAV8-based CRISPR reagents were injected into the tibialis anterior muscles of *mdx* mice to improve muscle function. At eight weeks post-injection, the muscles were harvested and analyzed for the deletion of exon 23 from the genomic DNA and mRNA, as well as for the expression of dystrophin protein. Droplet digital PCR (ddPCR) was used to quantify the percentage of modified alleles by separately amplifying unmodified or deleted DNA templates. Additionally, ddPCR showed that exon 23 was deleted in approximately 2% of all alleles in whole-muscle lysate. Western blotting of whole-muscle lysates showed substantial recovery of the dystrophin protein to approximately 8% of the normal level. Immunostaining revealed that approximately 67% of the myofibers expressed dystrophin. As little as 4% of the normal dystrophin expression level is sufficient to improve muscle function [126,127], and the present approach may be feasible as a potential therapy to treat DMD.

### 4.4. Neonatal Gene KO in the CNS

Neonatal ICV injections were used to deliver rAAV9 carrying sgRNAs directly into the CSF of neonatal mice (P0) to enable widespread transduction in the CNS [94,98]. AAV-PHP.B, an engineered rAAV9 variant, has been shown to broadly transduce CNS tissues with very high efficiency upon systemic delivery, much better than rAAV9 [128,129].

Hana et al. [122] used CRISPR/Cas9 to disrupt a neuron-specific gene (*NeuN*) and optimized key parameters to achieve effective gene KO in the CNS of postnatal mice (P0). To enhance the efficiency of CRISPR, AAV-PHP.B (2 × 10^11^ GCs in 4 μL) was used to deliver this sgRNA in Cas9 mice (Tg mice expressing Cas9 systemically) via neonatal ICV injection, based on the method of Kim et al. [97]. This approach resulted in a 99% biallelic indel rate in the transduced cells, leading to a greater than 70% reduction in total NeuN proteins in the cortex, hippocampus, and spinal cord. This work will be beneficial for the rapid assessment of the function of a GOI in vivo, as well as of its role in the pathology and etiology of CNS diseases.

### 4.5. Clinical Trials Using CRISPR/Cas9-Based Genome Editing

To date, several CRISPR/Cas9-based clinical trials have been attempted for the treatment of relapsed/refractory B-cell acute lymphoblastic leukemia [130], transfusion-dependent β-thalassemia [131], refractory B-cell leukemia [132], and sickle-cell disease [133]. As previously explained, although CRISPR/Cas9 is a promising tool for gene therapy, it is always associated with off-target mutagenesis. Therefore, further studies on the efficacy and safety of CRISPR/Cas9 are required [134]. Furthermore, an efficient gene delivery tool with minimal toxicity and immunogenicity is required [135]. The accumulated data will pave the way for coordinated clinical trials using CRISPR/Cas9 in neonates, children, and adults for the treatment of various genetic diseases.

## 5. Advantages and Limitations of Using Newborns

Neonates have many advantages over adults for performing in vivo somatic genetic engineering. The advantages and limitations of using newborn vs. adult animals are summarized in Table 3.

The most important aspect of experiments involving newborns is anesthesia. Neonates can be easily anesthetized by inducing hypothermia within 5–10 min when placed on ice for 5–10 min. In this case, administration of an anesthetic reagent is not needed. This condition can be maintained for at least 30 min during this period of genetic manipulation, such as the injection of nucleic acids, and in vivo EP towards the injected sites may be possible. After this treatment, they can recover when they are moved to the physiological condition (37 °C) and then nursed by the mother without any noticeable problems.

In the first few days after birth, the immune system in neonates is not well developed; therefore, it is theoretically possible to transplant human-derived cells, such as tumors and stem cells [136]. No immune response was detected when repeated injections of nucleic acids were administered 4 weeks after the first i.m. injection at P5 [107]. The immaturity of immunity in neonates can be reduced when the CRISPR system is applied to adult rodents. According to Wang et al. [137], Cas9 expressed as a foreign protein in a gene replacement therapy trial elicited pathological immune responses. However, systemic delivery of rAAV vectors in newborns helps mitigate potential immunological adverse events [138]. Exposure of newborn pups to AAV-encoded proteins probably induces tolerance to these proteins, thereby circumventing the toxicity caused by destructive adaptive immune responses.

The number of reagents introduced into neonates is smaller than that required for adults, which is beneficial for researchers who want to perform experiments in a cost-effective manner. Furthermore, at neonatal stages, when the development of the BBB is not fully established, intravenously administered substances can be transferred to cells in the CNS beyond the BBB [110]. This facilitates the genetic manipulation of these cells in the CNS using viral or non-viral vectors.

Intracerebral injections can be performed easily, without the need for special surgical interventions. Viruses, particularly rAAVs, have been extensively used for the transduction of CNS cells, but are difficult to generate at high titers, and have limitations in terms of insert and promoter size. To avoid the use of viral vectors, intracerebral injection of plasmid DNA and subsequent in vivo EP may be feasible for transfecting CNS cells, because in vivo EP is widely recognized as a powerful tool to deliver non-viral DNA, such as plasmids, into rodents [46]. No prominent post-EP effects on the survival or behavior of individuals have been reported.

Neonates have higher stem/progenitor cell ratios; therefore, transduction into stem/progenitor cells may occur more efficiently in neonates than in adults. Notably, almost all CNS neurons in the newborn pups ceased to proliferate. NSCs are located in the walls of the lateral ventricle and are involved in the generation of granular and PG cells during postnatal OB neurogenesis [92]. Intracerebral injection of nucleic acids into the lateral ventricles of newborn pups has been performed to explore the mechanism of generating neuronal cells from NSCs [88,89,90,92,96].

Furthermore, neonatal hepatocytes exhibit active proliferation; however, proliferation ceases in the adult stage. Lisjak et al. [121] suggested caution when F9-KO newborn mice are subjected to tail vein injection of rAAV carrying human *F9* cDNA to rescue the bleeding phenotype. The treated mice had detectable levels of F9 in the plasma and liver. However, the F9 levels were not sufficient to significantly ameliorate the bleeding phenotype in the tail clip assay. This is due to the nature of juvenile hepatocytes, which show active cellular proliferation; during active cell proliferation, the rAAV vectors introduced inside a cell are lost. Based on these experiments, Lisjak et al. [121] decided to use the CRISPR/SaCas9 platform, which could potentially be applied to the treatment of young patients with hemophilia. In contrast to juvenile hepatocytes, CNS neurons and skeletal muscle cells are non-proliferative. The introduction of transgenes (in the form of viral and non-viral vectors) into these cells results in long-term transgene expression because they are freely present in the cytoplasm as episomes. Thus, these cells are typical targets for CRISPR-based KI, KO, or transgene overexpression.

Neonatal skin appears to have a thinner epidermis than adult skin and is almost absent from dorsal hair skin, thereby facilitating skin-targeted genetic manipulation, as described by Titomirov et al. [65]. The skin plays a critical role in the formation of an intact epidermal barrier that separates the body from the environment. In addition to barrier formation, newborns have an immature cellular immune system that is essential for cutaneous defense against invasive bacterial disease [139]. In this context, an attempt to transfect neonatal epithelial skin cells may be important for assessing the function of epithelial biology as well as the mechanisms of the innate immune response.

Notably, there may be differences in the CRISPR-associated performance between newborns and adults when hepatocytes are selected as targets for somatic gene engineering. For example, Yang et al. [118] succeeded in rescuing lethal hyperammonemia from a chow diet after neonatal i.v. injection of rAAVs carrying CRISPR components, whereas gene correction through tail vein injection in adult *OTC*-deficient mice failed to rescue lethal hyperammonemia from a chow diet. When the treated adult mice were examined in more detail, larger deletions in the target *OTC* gene were found, which led to the ablation of residual expression of the endogenous *OTC* gene and diminished protein tolerance.

## 6. Conclusions

Traditional methods for modifying GOI expression through the production of Tg or gene-targeted mice have proven to be powerful strategies for the manipulation of gene expression, but they require time-consuming and expensive germline transgenesis. Alternative de novo approaches—such as i.v. injection of nucleic acids via the facial vein, or direct injection of nucleic acids and subsequent in vivo EP at the injection site using neonatal pups—yield faster results and are less costly. In addition to gene manipulation in neonatal pups, in utero gene manipulation at the fetal stage has been extensively performed to create individuals with in vivo somatic mutations and to explore fetal gene therapy approaches [7]. However, in utero manipulation requires invasive surgery, special interventions, and extensive care. This is in contrast to handling newborns, where no special interventions or techniques are needed. Because of the ease of gene delivery, enrichment of juvenile cells (stem cells), and convenient simple anesthesia, neonatal rodents are ideal experimental targets for exploring gene function and the effects of these gene products on the behavior of adult individuals.

More importantly, the manipulation of gene expression in the early developmental stages of neonates would be particularly valuable for preventing potential lethality or morbid perinatal diseases after birth. The small size of neonates, generation of immune tolerance, and delivery of a higher effective dose may be beneficial for this approach. In particular, immune tolerance allows for the re-administration of therapeutic Tg proteins in later developmental stages.

CRISPR-based genome editing systems using the Cas9 protein and sgRNA are powerful tools to regulate the gene expression of GOIs in vivo and can be useful for achieving gene correction at a target mutated locus in the gene therapy field. Concerns remain regarding safer delivery options (which are currently being extensively explored), possible off-target problems, and the potential for bacterial Cas9 proteins and viral vectors to induce an immune response when applied for clinical use in humans. In this context, the recent novel technologies called “prime or base editing” systems [12,13,14,15,16,17]—froms of CRISPR/Cas9-based genome editing technology allowing for the introduction of point mutations in the DNA without generating DSBs—would be safer than the pre-existing traditional CRISPR/Cas9, which depends on the generation of DSBs.

## Figures and Tables

**Table 1:** Summary of the production of gene-engineered newborn animals between the years 1991 and 2021.

Route and Method of Gene Delivery	Type of Nucleic Acid(s) Introduced	Outcome	Target Gene or Gene of Interest (GOI) Introduced	References
Facial-vein-mediated injection (also called i.v. injection)	Recombinant adeno-associated virus (rAAV)	Single injection of rAAV carrying human *beta-glucuronidase* (*GUSB*) cDNA into mucopolysaccharidosis type VII (MPS VII) model mice resulted in the expression of GUSB in most organs for 16 weeks and decreased or complete prevention of lysosomal storage; particularly, cells in the CNS were cleared of disease, suggesting viral infection beyond the blood–brain barrier (BBB)	*GUSB*	Daly et al., 1999 [66]
Facial-vein-mediated injection (also called i.v. injection)	Retroviral vector (RV)	Neonatal injection of RV carrying canine *GUSB* cDNA into dogs with MPS VII resulted in clonal expansion of hepatocytes and secretion of active GUSB into serum, along with clinical improvements; this is the first successful application of gene therapy in preventing a lysosomal storage disease in a large animal	*GUSB*	Ponder et al. 2002 [67]
Facial-vein-mediated injection (also called i.v. injection)	RV	Neonatal injection of RV carrying canine *GUSB* cDNA into MPS VII mice resulted in transduction of 6 to 35% of hepatocytes, which secreted GUSB into the blood; the secreted enzyme was taken up by other tissues and reduced the histopathological evidence of lysosomal storage in the liver, spleen, kidneys, small intestine, neurons, and glial cells	*GUSB*	Xu et al., 2002 [68]
Facial-vein-mediated injection (also called i.v. injection)	Adenoviral vector (AD)	Neonatal single injection of AD carrying human *GUSB* cDNA into MPS VII mice resulted in the recovery of more than 20% of GUSB activity in the brain, leading to the prevention of lysosomal storage, and lacking characteristic facial skeletal deformities associated with bone deformity, mental retardation, corneal clouding, and retinal degeneration	GUSB	Kamata et al., 2003 [69]
Facial-vein-mediated injection (also called i.v. injection)	rAAV2	Neonatal i.v. injection of rAAV2 carrying human iduronidase (*IDUA*) cDNA was performed using newborns with mucopolysaccharidosis type I (MPS I) to determine the potential for the gene delivery approach; high levels of IDUA activity were present in the treated animals and persisted for the 5-month duration of the study; successful correction of metabolic, craniofacial, and neurological abnormalities in MPS I mice was achieved	*IDUA*	Hartung et al., 2004 [70]
Facial-vein-mediated injection (also called i.v. injection)	Lentiviral vector (LV)	Single injection of LV into MPS I model mice resulted in the expression of IDUA activity, decreased glycosaminoglycan (GAG) storage, prevention of skeletal abnormalities, a more normal gross appearance, and improved survival; most strikingly, transduction of neurons at high levels was prominent	IDUA	Kobayashi et al., 2005 [71]
Peripheral (tail vein) i.v. injection	rAAV2/8 rAAV2/9	Both rAAV2/8 and rAAV2/9 vectors caused substantial transduction in the heart, skeletal muscle, and pancreas; importantly, rAAV2/9 transduced myocardium 5–10-fold higher than rAAV2/8, resulting in over 80% cardiomyocyte transduction, and suggesting that rAAV2/9 is superior to rAAV2/8 specifically for cardiac gene delivery	*F9* *nlslacZ* *lacZ*	Inagaki et al., 2006 [72]
Facial-vein-mediated injection (also called i.v. injection)	Sleeping Beauty (SB) transposon	Newborn mice were first i.p. injected with mannitol, and 1 h later luciferase-transposon DNA conjugated with polyethylenimine (PEI) was injected into the lateral ventricle; the treated animals showed significantly higher luciferase expression one month after gene delivery, suggesting chromosomal integration of the luciferase transgene and the usefulness of mannitol pretreatment combined with transposon-mediated gene transfer for long-term gene expression in the mammalian brain	*L* *uciferase*	Demorest et al., 2006 [58]
Facial-vein-mediated injection (also called i.v. injection)	rAAV2/1 rAAV2/8 rAAV2/9	When comparing rAAV2/1 with rAAV2/8 and the newer rAAV2/9 vectors for the transduction of skeletal muscle, both rAAV2/8 and rAAV2/9 were able to transduce myocardium at approximately 20- and 200-fold (respectively) higher levels than rAAV2/1; thus, rAAV2/9 is more readily able to cross the vasculature and leads to preferential cardiac transduction in vivo, efficiently transducing cardiac tissue	*lacZ*	Pacak et al., 2006 [73]
Facial-vein- or external jugular-vein-mediated injection (also called i.v. injection)	-	Two techniques (injection via the external jugular vein, and via the superficial temporal vein) for i.v. injection in neonatal mice from birth to 6 days of age were described; both allow the injection of a variety of substances, including cells, medications, toxins, and cytokines, and both permit serial injections	-	Kienstra et al., 2007 [60]
Facial-vein-mediated or intraperitoneal (i.p.) injection	rAAV8	Neonatal administration of rAAV8 by i.v. or i.p. injection was performed to test whether lower motor neurons (LMNs) are transduced, indicating that dorsal root ganglion transduction occurs across all timepoints and injection routes	*GFP*	Foust et al. 2008 [74]
Facial-vein-mediated injection (also called i.v. injection)	Helper-dependent adenoviral vector (AD)	Intravenous (i.v.) injection of AV expressing human factor 8 (F8) to neonatal hemophilia A (HA)-knockout (KO) mice resulted in the production of high levels of F8, and its expression lasted until >1 y of age, which is associated with correction of HA and tolerance to human F8	*F8*	Hu et al., 2011 [75]
Facial-vein-mediated injection (also called i.v. injection)	rAAV2/9	Neonatal administration of rAAV2/9 produced global delivery to the central (i.e., brain, spinal cord, and all layers of the retina) and peripheral nervous system (i.e., myenteric plexus and innervating nerves), which can provide a therapeutic strategy for the treatment of early lethal genetic diseases, such as Gaucher disease	*GFP*	Rahim et al. 2011 [76]
Facial-vein-mediated injection (also called i.v. injection)	rAAVrh10	Neonatal co-injection of two AAVrh10s (in which one had a heavy chain and the other a light chain for F8) into mice with HA resulted in long-term correction and avoidance of immune responses in the AAV-F8-treated mice	*F8*	Hu and Lipshutz 2012 [77]
i.v.-mediated injection	rAAV9 carrying mutating capsid surface tyrosines	A double-tyrosine mutant of rAAV9 significantly enhanced gene delivery to the CNS and retina, and that gene expression could be restricted to rod photoreceptor cells by incorporating a rhodopsin promoter, which may provide a new methodology for the development of retinal gene therapies or the creation of animal models of neurodegenerative diseases	*GFP*	Dalkara et al., 2012 [78]
Facial-vein-mediated injection (also called i.v. injection)	Self-complementary (sc) rAAV9	Neonatal i.v. injection of rAAV9 resulted in gene transfer to all layers of the retina (including retinal pigment epithelium cells, photoreceptors, bipolar cells, Müller cells, and retinal ganglion cells) in adult mice; in particular, the cells on the inner side of the retina were transduced with the highest efficiency, suggesting that this vector serotype is able to cross mature blood–eye barriers	*GFP*	Bemelmans et al., 2013 [79]
Facial-vein-mediated injection (also called i.v. injection)	rAAV2/1 rAAV2/5 rAAV2/6 rAAV2/8 rAAV2/9	Intravenous (i.v.) injection of AAV9 resulted in the transduction of 25–57% of enteric nervous system (ENS) myenteric neurons; AAV9 transduction in enteric glia was very low compared to CNS astrocytes; AAV8 resulted in comparable transduction in neonatal mice to AAV9, while AAV1, -5, and -6 were less efficient, suggesting that systemic AAV9 has a high affinity for peripheral neural tissue and will be useful for future therapeutic development and basic studies of the ENS	*GFP*	Gombash et al., 2014 [80]
Facial-vein-mediated injection (also called i.v. injection)	Single-stranded (ss) and scAAV9	Extensive GFP expression was observed in organs throughout the body, with the epithelial and muscle cells being particularly well transduced, suggesting that AAV9 can potentially be used for clinical systemic gene therapy protocols	*GFP*	Mattar et al., 2015 [81]
Facial-vein-mediated injection (also called i.v. injection)	rAAV2/8 rAAV2/9	Both rAAV2/8 and rAAV2/9 showed equal potential in transducing the ENS, with 25–30% of the neurons expressing EGFP; all enteric neuron subtypes, but not glia, expressed the reporter protein; these findings will be used for novel preclinical applications aimed at manipulating and imaging the ENS in the short term, and for gene therapy in the longer term	*EGFP*	Buckinx et al., 2016 [82]
Facial-vein-mediated injection (also called i.v. injection)	rAAV2/9	Resulted in binaural transduction of inner hair cells, spiral ganglion neurons, and vestibular hair cells; transduction efficiency increased in a dose-dependent manner; inner hair cells were transduced in an apex-to-base gradient, with transduction reaching 96% in the apical turn; intravenous delivery of rAAV2/9 represents a novel and atraumatic technique for inner-ear transgene delivery in early postnatal mice	*EGFP*	Shibata et al., 2017 [83]
Facial-vein-mediated injection (also called i.v. injection) or intracerebral injection	Foamy virus (PFV)	Systemic PFV vector delivery to neonatal mice gave transgene expression in the heart, xiphisternum, liver, pancreas, and gut, whereas intracranial administration produced brain expression; transgene expression was highly localized to the hippocampal architecture, despite vector delivery being administered to the lateral ventricle; PFV can be used for neonatal gene delivery targeted to hippocampal neurons, for gene therapy of neurological disorders	*EGFP*	Counsell et al., 2018 [84]
Facial-vein-mediated injection (also called i.v. injection)	AAVs	Intravenous (i.v.) administration can achieve widespread delivery of rAAVs to the CNS, which are considered to be promising therapeutic tools for treating genetic defects of the CNS, due to their excellent safety profile and ability to cross the BBB	*-*	Gessler et al., 2019 [85]
Facial-vein-mediated injection (also called i.v. injection)	rAAV8	Intravenous (i.v.) administration can achieve widespread gene expression in the central and peripheral nervous system, liver, kidneys, and skeletal muscle; i.v. injection of rAAV8 carrying a spleen focus-forming virus (SFFV) promoter and nuclear factor-κB (NF-κB) binding sequence for bioluminescence and biosensor evaluation resulted in a 10-fold increase in *luc* expression after single administration of lipopolysaccharide (LPS), and whole-body bioluminescence persisted for up to 240 days	*GFP* *luc*	Karda et al., 2020 [6]
Intracerebral injection	rAAV2	Gene transfer throughout the CNS was achieved without germline transmission, and gene expression lasted for at least 1 year	*GUSB*	Passini and Wolfe 2001 [86]
Intracerebral injection	rAAV1 rAAV2 rAAV5	The 0gene delivery pattern after neonatal intracerebral injection was assessed using different rAAV serotypes; consequently, rAAV5 showed very limited brain transduction, even though it has different transduction patterns than rAAV2; in contrast, rAAV1 vectors showed robust widespread transduction; in the majority of structures, rAAV1 transduced many more cells than rAAV2	*GUSB*	Passini et al., 2003 [87]
Intracerebral injection and subsequent in vivo electroporation (EP)	Plasmid DNA	Intraventricular injection of DNA followed by EP induced strong expression of transgenes in the radial glia, neuronal precursors and neurons of the olfactory system; overexpression of the cell-cycle inhibitor p21 resulted in interference with the proliferation of neural stem cells	*hNCAM-140* *p21*	Boutin et al., 2008 [88]
Intracerebral injection and subsequent in vivo EP	Plasmid DNA	Neonatal intracerebral injection was performed to determine how neuroprogenitors in the subventricular zone (SVZ) give rise to neuroblasts that migrate along the rostral migratory stream (RMS); labeling was found in all classes of interneurons in the olfactory bulb (OB), persisted to adulthood, and had no adverse effects	*EGFP*	Chesler et al., 2008 [89]
Intracerebral injection	rAAV8 LV	Region-specific recombination of a “stop-floxed” *Rosa26* reporter allele was achieved upon targeted injection of rAAV vectors expressing Cre-recombinase (Cre); utilizing LV, efficient transduction of neuroprogenitors in the SVZ occurred and, as a result, approximately 20% of labeled migrating neuroblasts were generated along the RMS into the OB	*Cre*	Pilpel et al., 2009 [90]
Intracerebral injection and subsequent in vivo EP	Plasmid DNA	Neonatal non-ventricular injection into deep cortical layers or the striatum region, followed by EP, was performed to create a local expression pattern in the area of interest and in situ transfection of non-migratory cell types, e.g., cortical astrocytes, which may be used for two-photon in vivo imaging	*EGFP*	Molotkov et al., 2010 [91]
Intracerebral injection and subsequent in vivo EP	Plasmid DNA	Improvement of the EP technique allowing for targeted transgene delivery to specific walls of the lateral ventricle, accurately and reproducibly, was performed to trace perinatal neural stem cells, which successfully enabled fate mapping of the progeny of RGCs	*EGFP*	Fernandez et al., 2011 [92]
Intracerebral injection	rAAV8	Neonatal intra-cerebral injection of viral vectors into the hindbrain enables postnatal dendritic maturation in cerebellar Purkinje neurons for in vivo imaging of mature Purkinje neurons at a resolution sufficient for complete analytical reconstruction	*YFP* *tdTomato* *iCre* tTA	Kim et al., 2013 [93]
Intracerebral injection	rAAV2/1 rAAV2/2 rAAV2/5 rAAV2/7 rAAV2/8 rAAV2/9	The effects of the timing of the injection on the rAAV tropism and biodistribution of six commonly used rAAVs after neonatal intracerebral gene delivery was assessed; consequently, rAAV2/8 and 2/9 resulted in the most widespread distribution in the brain; injection on neonatal day P0 resulted in mostly neuronal transduction, whereas administration at later periods of development resulted in more non-neuronal transduction; rAAV2/5 showed widespread transduction of astrocytes irrespective of the time of injection; none of the serotypes tested showed any microglial transduction	*EGFP*	Chakrabarty et al., 2013 [94]
Intracerebral injection	LV	Neonatal intracerebral injection of LV carrying the β-galactocerebrosidase (*GALC*) gene in the external capsule of twitcher mice, a severe model of globoid cell leukodystrophy, resulted in the restoration of GALC activity in the whole CNS of treated mice as early as 8 days post-injection; this approach will be useful for neonatal LV-mediated intracerebral gene therapy	*GALC* *GFP*	Lattanzi et al., 2014 [95]
Intracerebral injection and subsequent in vivo EP	Plasmid DNA	Neonatal intracerebral injection of plasmid and subsequent in vivo EP were performed to analyze the RMS and postnatal OB neurogenesis; consequently, GFP-positive cells in the dentate gyrus (DG) were observed to extend branched dendrites and long axons into the molecular layer and the hilus; the expression of GFP in these neurons was sustained for at least 9 months	*GFP*	Ito et al., 2014 [96]
Intracerebral injection	rAAV8	Intraventricular injection of rAAV8 within the first 24 h after birth resulted in widespread transduction of neurons throughout the brain; expression began within days of the injection and persisted for the lifetime of the animal; this versatile manipulation enables studies ranging from early postnatal brain development to aging and degeneration in the adult	*YFP* *tdTomato*	Kim et al., 2014 [97]
Intracerebral injection	rAAV2/1 rAAVDJ8 rAAV9	Tropism of rAAV2/1, rAAVDJ8, and rAAV9 throughout different brain regions and cell types was assessed through neonatal intracerebral injection; rAAV2/1 infections were more prevalent in the cortical layers but penetrated to the midbrain less than rAAVDJ8 and rAAV9; rAAVDJ8 displayed more tropism in astrocytes compared to rAAV9 in the substantia nigra (SN) region	*GFP*	Hammond et al., 2017 [98]
Intracerebral injection	rAAV9 AAV-PHP.B AAV-PHP.eB	Neonatal intra-brain injection of rAAV9, AAV-PHP.B, and AAV-PHP.eB carrying CRISPR reagents was used to examine cell-type-specific gene ablation; consequently, AAV-PHP.B variants exhibited marked disruption of neuron-related genes, but only modest disruption of the astrocyte- or oligodendrocyte-specific genes was observed by all three AAV variants, which could facilitate profiling of AAV cellular tropism in the murine CNS	*NeuN* *GFAP* *MOG*	Torregrosa et al., 2021 [99]
Intraperitoneal (i.p.) injection	rAAV1 rAAV2 rAAV5 rAAV6 rAAV7 rAAV8	Neonatal single injection of various serotypes of rAAVs via i.p. or i.v. routes was performed; rAAV8 was the most efficient vector for crossing the blood vessel barrier to attain systemic gene transfer in both skeletal and cardiac muscles; rAAV1 and rAAV6, which demonstrated robust infection in skeletal muscle cells, were less effective in crossing the blood vessel barrier; gene expression persisted in the muscle and heart but diminished in the liver, which showed rapid cell division; this approach will be useful for muscle-directed systemic gene therapy	*GFP*	Wang et al., 2005 [100]
i.p. injection	Single-stranded AAV9 (ssAAV9) carrying short hairpin RNA (shRNA)	Neonatal i.p. injection of shRNA-ssAAV9 resulted in ~80% reduction in target mRNA in the DRG, along with 75% suppression of the protein; the suppression effect lasted for more than 3 months; this approach may be helpful for elucidating the mechanisms of pain and sensory ganglionopathies	*Sod1*	Machida et al., 2013 [64]
i.p. and i.v. injection	rAAV8	Neonatal i.p. or i.v. injection of rAAV8 through entry into the nervous system was performed to target lower motor neurons (LMNs) for future gene therapy against spinal muscular atrophy and amyotrophic lateral sclerosis; consequently, spinal cords were positively transduced; furthermore, fibers in the dorsal horns and columns were labeled, indicating dorsal root ganglion transduction with these techniques	*GFP*	Foust et al., 2008 [74]
i.v. injection via the retro-orbital sinus	RV	Neonatal retro-orbital-sinus-mediated injection of an RV vector carrying *F8* cDNA into *F8*-deficient mice resulted in successful correction of HA	*F* *8*	Vanden Driessche et al., 1999 [101]
Injection into the subretinal space and subsequent in vivo EP	Plasmid DNA	Neonatal injection of plasmid DNA into the subretinal space of mice, and subsequent in vivo EP, resulted in successful transfection of retinal cells; transfection of plasmid DNA carrying RNAi resulted in a knockdown phenotype of a target gene’s expression, which was similar to the phenotype shown in KO mice	*GFP*	Matsuda and Cepko 2004 [102]
Injection into the subretinal space and subsequent in vivo EP	Plasmid DNA	Conditional temporal and spatial regulation of gene expression in the retinas of postnatal rats was assessed using Cre/*loxP*-mediated inducible expression vectors and 4-hydroxytamoxifen treatment, which enables conditional activation of Cre recombinase; transgene expression was successfully induced in a cell-type- and time-specific manner	*CreERT2* *DsRed* *GFP*	Matsuda and Cepko 2007 [103]
Hydrodynamics-based gene delivery (HGD) via the retro-orbital sinus	Plasmid DNA	High levels of gene expression in the hepatocytes of neonatal mice were achieved, which will provide a way to perform gene delivery to animals that are difficult to inject via the tail vein; it will also be beneficial for exploring gene function and treating genetic disease	*Luciferase*	Yan et al., 2012 [104]
i.v. injection via the retro-orbital sinus	First-generation AD vector	The highest AD vector genome copy numbers and transgene expression were found in the neonatal liver; the neonatal heart exhibited the second-highest levels of transgene expression among the organs examined; no apparent hepatotoxicity was observed in neonatal mice; these findings may be helpful for performing gene therapy using AD vectors in neonates	*Luciferase*	Iizuka et al., 2015 [105]
i.v. injection via the retro-orbital sinus	rAAV9	Showing the benefits of i.v. injection via the retro-orbital sinus as an effective route of rAAV9-mediated gene delivery in neonates	*Cre*	Prabhakar et al., 2021 [59]
Intramuscular (i.m.) injection	rAAV	Neonatal i.m. injection of an rAAV vector carrying human *GUSB* cDNA into MPS VII mice resulted in high-level intramuscular GUSB expression as early as 2 weeks of age, and for at least 16 weeks; GUSB activity was detected in both the liver and spleen at later timepoints, indicating that rAAV vectors can successfully infect neonatal muscle and persist through the rapid growth phase following birth	*GUSB*	Daly et al., 1999 [106]
i.m. injection	Plasmid DNA	Neonatal intramuscular injection of plasmid DNA into hypercholesterolemic mice (*ApoE* KO mice) resulted in a reduction in the incidence of severe hypercholesterolemia; notably, when naked DNA was administrated early, no immune response was generated against the human APOE3, allowing for repeated administrations; this approach will be useful for treating many genetic childhood diseases where early administration is required to prevent developmental damage	*APOE3*	Signori et al., 2007 [107]
Injection into the skin and subsequent in vivo EP	Plasmid DNA	Subcutaneous injection of two plasmid DNAs (one with a neomycin-resistance gene (*neo*) and the other with an immortalizing gene) into the skin cells of newborn mice (at P1-3), followed by subsequent in vivo EP, resulted in the generation of stably transfected fibroblasts	*neo*	Titomirov et al., 1991 [65]

**Table 2:** Summary of the production of genome-edited newborn animals between the years 2016 and 2022.

Route and Method of Gene Delivery	Type of Nucleic Acid(s) Introduced	Outcome	Target Gene or Gene of Interest (GOI) Introduced	References
Facial-vein-mediated injection (also called i.v. injection)	Dual rAAV (one with the *Cas9* gene and the other with gRNA/donor DNA)	Neonatal i.v. injection of dual AAVs into *OTC*-deficient mice was performed to correct metabolic liver disease caused by lethal hyperammonemia; consequently, 10% of hepatocytes were restored, and reduced survival after feeding with a chow diet was avoided	*OTC*	Yang et al., 2016 [118]
Facial-vein-mediated injection (also called i.v. injection)	Dual rAAV8 (one with SaCas9/sgRNA and the other with donor DNA)	Neonatal i.v. injection of an SaCas9/sgRNA-expressing rAAV8 vector into wild-type mice resulted in mutations in the *F9* gene in hepatocytes, sufficiently developing HB; also, it was possible to generate HDR-based correction of the mutated *F9* gene in HB model mice; this approach will provide a flexible approach to induce DSB-mediated mutations in target genes in hepatocytes, and also to cure congenital hemorrhagic disease	*F9*	Ohmori et al., 2017 [119]
Facial-vein-mediated injection (also called i.v. injection)	Dual rAAV8 (one with *SaCas9* and the other with gRNA/donor DNA containing *F9* cDNA carrying a hyperactive *F9* Padua mutation	Neonatal i.v. injection of dual rAAVs into *F9* KO mice resulted in expression of F9 at a normal level over 8 months, suggesting the use of the CRISPR approach to achieve lifelong expression of therapeutic proteins	*F9*	Wang et al., 2019 [120]
Facial-vein-mediated injection (also called i.v. injection)	rAAV8 carrying donor DNA and rAAV8 carrying *SaCas9* and sgRNA	Neonatal i.v. injection of dual rAAV8 vectors into *F9* KO mice resulted in the stable expression of human F9, reaching up to 150% of the human levels, showing a clotting capacity comparable to wild-type animals, and demonstrating the rescue of the disease phenotype	*F9*	Lisjak et al., 2022 [121]
Intracerebral injection	AAV-PHP.B carrying sgRNA	Neonatal intracerebral injection of AAV-PHP.B carrying sgRNA into Cas9 mice resulted in a 99.4% rate of biallelic indels in the transduced cells, leading to a more than 70% reduction in the quantity of total NeuN proteins in the cortex, hippocampus, and spinal cord	*NeuN*	Hana et al., 2021 [122]
i.p. injection	Dual rAAV9 carrying CRISPR components	To correct Duchenne muscular dystrophy (DMD) by skipping mutant dystrophin exons in postnatal muscle tissue in vivo, rAAV9 carrying CRISPR/Cas9 components was i.p. injected into neonatal *mdx* mice (P1), a model of DMD; as a result, dystrophin protein expression was observed in cardiac and skeletal muscle to varying degrees, and expression increased from 3 to 12 weeks after te injection	*Dystrophin*	Long et al., 2016 [123]
i.m. injection	Dual rAAV8 carrying CRISPR components	Neonatal i.m. injection of rAAV8 carrying CRISPR components into an *mdx* mouse model of DMD was performed to improve muscle function; removal of mutated exon 23 from the dystrophin gene resulted in expression of the modified dystrophin gene, partial recovery of functional dystrophin protein in skeletal myofibers and cardiac muscle, and significant enhancement of muscle force, suggesting that this approach is useful as a potential therapy to treat DMD	*Dystrophin*	Nelson et al., 2016 [124]
i.m. injection	Dual rAAV9 carrying CRISPR components	To test whether the removal of one or more exons from the mutated transcript in DMD-related dystrophin could produce an in-frame mRNA and a truncated but still functional protein, dual rAAVs carrying CRISPR/Cas9 components were subjected to i.m. injection into neonatal *mdx* model mice; as a result, in the treated mice, restoration of dystrophin expression was observed in myofibers, cardiomyocytes, and muscle stem cells	*Dystrophin*	Tabebordbar et al., 2016 [125]

**Table 3:** Advantages and limitations of using newborn vs. adult animals for in vivo somatic genetic manipulation.

Procedure or Property	Newborns	Adults
Anesthesia	Relatively easy, because newborn pups are prone to hypothermia after being placed on a chilled paper towel on top of ice for 30–60 s; therefore, there is no need for an anesthetic agent, but the surgical procedure must be performed within ~30 min	Requires administration of an anesthetic agent or isoflurane; surgical procedure can be performed for more than 1 h
Immunological tolerance	The immune system of newborn pups at P0 to P7 (whose condition is also called “immune immaturity”) is not well established; therefore, it is possible to transplant xenogenic cells such as human-derived cells	The immune system is already tightly established; therefore, it is impossible to transplant xenogenic cells such as human-derived cells
Intravenous (i.v.) injection	Relatively easy; a small amount of solution (up to 50 μL) can be injected via the facial vein; therefore, the reagents required can be reduced at a minimal level; however, the appearance of the facial vein is temporal, as it disappears up to 6 days after birth; tail vein injection becomes possible from P15 onward; therefore, it may be theoretically impossible to perform i.v. injection during the periods of P7 to P14	Relatively easy; relatively large amounts of solution (~1 mL) are generally required; therefore, the reagents used appear to be costly
Gene delivery beyond the blood–brain barrier (BBB) after i.v. injection	Due to the incomplete development of the BBB at this stage, small molecules such as recombinant adeno-associated viruses (rAAVs) injected exogenously can easily penetrate into cells of the central nervous system (CNS) via the BBB	Large molecules cannot be transferred inside a brain, since the established BBB does not allow penetration beyond the BBB
Intracerebral injection	Relatively easy; requires peeling of the skin and skull with fine forceps prior to the insertion of a capillary pipette under a dissecting microscope	It requires drilling of a skull prior to the insertion of the capillary pipette, which is laborious and time-consuming
Retro-orbital-sinus-mediated injection	Relatively easy	Relatively easy; repeated treatment is possible
Manipulation of internal organ	Relatively difficult, because organs are too small and fragile; therefore, their handling must be carried out under observation using a dissecting microscope, requiring special caution upon surgical treatment and skin closure after surgery, because the skin itself is thin and labile	Relatively easy; manipulation of internal organs can be performed during their exposure onto the skin; also, skin closure is easy after surgery

## Data Availability

Not applicable.

## Abbreviations

AAV	Adeno-associated virus
ABEs	Adenine base editors
AD	Adenoviral vector
APOE3	Apolipoprotein E3
BBB	Blood–brain barrier
BEs	Base editors
Cas9	CRISPR-associated protein 9
CBEs	Cytosine base editors
CNS	Central nervous system
CRISPR	Clustered Regularly Interspaced Short Palindromic Repeats
dCas9	Dead Cas9
ddPCR	Droplet digital PCR
DG	Dentate gyrus
DMD	Duchenne muscular dystrophy
DRG	Dorsal root ganglia
DSB	Double-stranded DNA break
EGFP	Enhanced green fluorescent protein
ENS	Enteric nervous system
EP	Electroporation
ER	Estrogen receptor
ERT2	Mutated ligand-binding domain of ER
ET	Egg transfer
F8 (9)	Factor 8 (9)
GAG	Glycosaminoglycan
GALC	β-Galactocerebrosidase
GCs	Genome copies
GFP	Green fluorescent protein
GOI	Gene of interest
GONAD/*i*-GONAD	Genome Editing via Oviductal Nucleic Acids Delivery/improved GONAD
gRNA	Guide RNA
GUSB	β-Glucuronidase
HDR	Homology-directed repair
HA	Hemophilia A
HB	Hemophilia B
HGD	Hydrodynamics-based gene delivery
ICV	Intracerebroventricular
IDUA	α-L-iduronidase
i.m.	Intramuscular
i.p.	Intraperitoneal
IT	Intrathecal
ITRs	Inverted terminal repeats
i.v.	Intravenous
KI	Knock-in
KO	Knockout
LMNs	Lower motor neurons
LPS	Lipopolysaccharide
LV	Lentivirus
MMEJ	Microhomology-mediated end-joining
MPS I	Mucopolysaccharidosis type I
MPS VII	Mucopolysaccharidosis type VII
NCAM (hNCAM-140)	Neural cell adhesion molecule
nCas9	Cas9 nickase
NF-*κ*B	Nuclear factor-*κ*B
NHEJ	Non-homologous end-joining
NSCs	Neural stem cells
OB	Olfactory bulb
4OHT	4-Hydroxytamoxifen
OTC	Ornithine transcarbamylase
PAM	Protospacer adjacent motif
PBS	Phosphate-buffered saline
PEs	Prime editors
pegRNA	Prime editing guide RNA
PFV	Foamy virus
PG	Periglomerular
RGCs	Radial glial cells
RMS	Rostral migratory stream
RNAi	RNA interference
RNP	Ribonucleoprotein
RT	Reverse transcriptase
RV	Retrovirus
rAAV	Recombinant adeno-associated virus
SaCas9	*Staphylococcus aureus*-derived Cas9
SB	Sleeping Beauty
SFFV	Spleen focus-forming virus
sgRNA	Single-guide RNA
shRNA	Short hairpin RNA
siRNAs	Short interfering RNAs
SN	Substantia nigra
Sod1	Superoxide dismutase 1
SVZ	Subventricular zone
TadA	tRNA adenine deaminase
TBG	Thyroxine-binding globulin
Tg	Transgenic
